# Stress, physical activity, and mindfulness practices among youth amidst COVID-19

**DOI:** 10.3389/fspor.2024.1493729

**Published:** 2024-12-02

**Authors:** A. El Hajj, M. Noulhiane, B. Andrieu, Natacha Heutte, O. Sirost

**Affiliations:** ^1^ Sport Sciences Department, Univ Rouen Normandie, CETAPS, Rouen, France; ^2^ CEA-Neurospin, UNIACT - Université Paris Saclay, Department of Neuroscience, Gif-sur-Yvette, France; ^3^ INSERM U1141, Université Paris Cité, Paris, France; ^4^ Sport Sciences Department, Université Paris Cité, I3SP, Paris, France

**Keywords:** stress, wellness and mindfulness activities, physical activity, mental well-being, youth, university students

## Abstract

**Introduction:**

The COVID-19 pandemic has exacerbated stress and anxiety among young people, particularly university students, impacting their mental well-being and daily life. Given the rise in social isolation and economic uncertainty, the adoption of mindfulness practices such as sophrology, meditation, and yoga becomes essential for improving their mental health. This study aims to evaluate the impact of these practices on stress levels and their effect on engagement in physical activities among youths.

**Method:**

This study employed a mixed methods design to assess the impact of different wellness and mindfulness practices on stress levels and physical activity (PA) engagement among university students. Quantitative data were gathered electronically from 218 students at the University of Rouen and the university of Paris-Cité through validated questionnaires, including custom tools on sports and mindfulness practices and the Perceived Stress Scale (PSS). The sample consisted mainly of young adults aged 18 to 35, with a majority of 170 female and 48 male participants. Qualitative insights were gathered through semi-structured interviews with three directors of the university's sports and physical activities department (SUAPS), as well as four wellness program instructors. Data analysis involved statistical techniques using Statistical Analysis System (SAS) software for quantitative data, while qualitative data were analyzed thematically using Sphinx software, a tool for textual analysis. Ethical approval for the study was obtained, and confidentiality of all participants was maintained throughout the research.

**Results and conclusion:**

The COVID-19 pandemic had significant effects on French university students’ PA, mental health, and stress levels. Lockdowns led to a decline in sports practices for many, while others adapted by increasing their engagement in fitness and wellness practices. The majority of participants (64.68%) reported moderate levels of stress. A significant correlation was found between motivations for mental health support and stress management, and higher levels of stress (*p* = 0.0000 and *p* = 0.0024, respectively). Regular participation in wellness activities was associated with lower stress (*p* = 0.0193). The findings reinforce the idea that incorporating wellness practices into educational environments can strengthen students’ mental resilience and overall well-being, equipping them with essential tools to effectively cope with future stressors.

## 1 Introduction

The impact of physical exercise on academic achievement has been thoroughly examined, demonstrating various beneficial effects. Regular physical exercise improves students’ cognitive functions, such as concentration and memory, while also honing their overall mental sharpness (1–4). Exercise has also been identified as a great stress reliever, allowing students to better cope with college demands and workloads (5–7). Furthermore, aside from boosting concentration, physical exercise promotes physical health and reduces absenteeism due to illness (8, 9). Participating in sports helps to develop vital skills like time management, discipline, and teamwork (10). Physical exercise can also improve academic motivation and help them to deal with worry, anxiety, stress or depression by boosting their self-esteem and confidence (11–13). The overall impact of exercise and physical activity (PA) on an individual's progress in education emphasizes the importance of exercising regularly, to enhance both academic and overall performance and achievement. Stress levels among youth, particularly among university students, are an increasing concern. Academic pressures such as high-grade expectations, tight deadlines, and juggling multiple subjects all have a significant impact on students’ mental health and well-being (14). Social dynamics are also important as students navigate their interactions with peers, family, and faculty. Furthermore, students often experience financial stress because they must pay for tuition and living expenses while also working part-time (15–18). The COVID-19 epidemic, particularly through the impact of lockdowns, has intensified these pressures, resulting in increased anxiety and lower overall well-being among youth (19, 20). The rapid shift to online education, along with social isolation and altered daily routines, has resulted in significantly increased stress and mental health difficulties. The loss of face-to-face communication, with the monotony of virtual classes, has resulted in students’ reduced attention span and engagement (21–23). The pandemic has also significantly reduced PA due to restrictions on movement and the closure of gyms and sport centers. This decline in PA has significantly impacted youth mental health, causing higher levels of anxiety and depression (24–26). Moreover, a major concern is the pandemic's influence on students’ cognitive capacities. Research findings indicate that pandemic-related stress and worry may have an impact on cognitive functions such as memory, focus, and decision-making. This disability might cause difficulty with learning and academic performance, exacerbating youth stress levels (27–30).

In France, university students’ participation rates in physical and sporting activities offer a crucial perspective for examining these issues. According to recent statistics, a significant number of university students engage in regular PAs, while there is substantial diversity depending on factors such as academic load, access to sports facilities, and individual preferences (31). According to the report from the National Observatory of Physical Activity and Sedentary Behavior (*Observatoire National de l'Activité Physique et de la Sédentarité*, ONAPS) and the National Association of STAPS Students (*Association Nationale des Étudiants en STAPS*, ANESTAPS) on the engagement in physical and sports activities (PSA) and sedentary behavior among university students in France, 41.8% of students participate in physical exercise at least five times per week, meeting the national PA recommendations. However, 10% of them are inactive, having not met the necessary levels of PA. On average, students engage in 10.5 h of PA each week, which includes a variety of activities ranging from light housekeeping to more intense sports (31). These findings are critical for contextualizing the favorable impacts of PSA on student well-being and academic outcomes in the French educational setting. For instance, students who maintain a routine of PAs report better mental health, decreased stress levels, and higher academic performance (7, 32). The availability of sports facilities and programs at colleges is critical in encouraging students to engage in physical exercise (33, 34). These efforts are crucial for supporting the physical and mental health of students, particularly in the context of the ongoing pandemic. On the other side, given the growing need for effective mental health strategies, the adoption of mindfulness techniques has become increasingly important for stress reduction and mental health improvement (35–37). Techniques such as sophrology, meditation, and yoga have become popular because of their accessibility and holistic benefits. Sophrology, which systematically combines relaxation techniques and imagery, aims to enhance mental clarity and calmness (38). Meditation promotes emotional stability cultivating a focused and quiet state of mind (39), whereas yoga combines physical postures, breath control, and meditation to improve physical fitness and inner tranquility (40). This paper thoroughly examines the impact of different mindfulness practices on stress levels and their influence on PA engagement among youths. By meticulously examining the effectiveness of mindfulness practices in reducing stress, this research aspires to provide robust, evidence-based recommendations for incorporating these practices into educational institutions’ curriculums. The ultimate goal is to advocate for a holistic and comprehensive approach to mental health, thereby supporting the well-being of university students both during and beyond the pandemic.

## 2 Method

### 2.1 Study design

In order to provide a comprehensive understanding of the impact of wellness and mindfulness practices on stress levels and student engagement in PAs, this study adopted a mixed-methods design combining both quantitative and qualitative approaches.

The study involved three key components:

• A systematic document analysis of existing literature on the effects of COVID-19 on the health behaviors of university students in France.
• Quantitative data collection through validated questionnaires assessing well-being, mindfulness, and sports practices.
• Qualitative data collection through semi-structured interviews with key stakeholders involved in wellness programs at two French universities.

As a result of this design, we were able to triangulate our findings, providing us with a useful insight into how wellness practices influence PA engagement and stress levels among youth.

### 2.2 Document analysis

Before collecting primary data, we conducted a systematic document analysis to contextualize our study and inform its design. The document analysis focused on scientific publications from 2020 to 2023, examining the effects the COVID-19 pandemic on the health behaviors of university students in France. The analysis aimed to identify the students’ situation in France during the pandemic and the existing patterns and gaps in the literature. This approach ensured that our research focused on relevant but previously unexplored areas.

The analysis covered both quantitative and qualitative studies sourced from PubMed, ScienceDirect, and BASE. Studies were selected based on predefined inclusion criteria, focusing on topics such as eating habits, PA levels, and quality of life among university students. Articles in both English and French were included to provide a comprehensive overview of the research landscape. The results of this analysis were used to shape the study's primary data collection instruments and to position our findings within the broader academic discourse on COVID-19's impact on student well-being.

### 2.3 Participants

A total of 218 participants took part in the quantitative study, and seven key stakeholders were interviewed for the qualitative study. The study population included:

• Quantitative study: 170 females and 48 males, with a mean age of 33 years (ranging from 18 to 75 years). Participants were students enrolled in wellness programs at SUAPS (university sports services) at the University of Rouen and the University of Paris-Cité. The majority (134 participants) were young adults aged 18–35, and most were undergraduates (71% pursuing bachelor’s degrees).
• Qualitative participants: Three SUAPS directors and four wellness instructors, specializing in Yoga, Pilates, and Sophrology, were interviewed based on their involvement in wellness programs and their expertise.

### 2.4 Data collection

#### 2.4.1 Quantitative data

Quantitative data were collected using validated questionnaires designed to measure stress levels and evaluate sports practices.

• Well-being and mindfulness practices: A custom-designed questionnaire tailored to capture participants’ engagement with mindfulness activities.
• Sports practices: A questionnaire evaluating the frequency and types of sports and PA.
• Perceived Stress Scale (PSS): A 10-item questionnaire used to measure the stress levels of participants.

The questionnaires were distributed electronically via email to students enrolled in SUAPS programs at the University of Rouen and the University of Paris-Cité. This method allowed for the collection of comprehensive data on students’ mindfulness practices, PA engagement, and stress levels.

#### 2.4.2 Qualitative data

To complement the quantitative data, qualitative data were gathered through semi-structured interviews with stakeholders directly involved in wellness programs at the two universities. Interviews were conducted with three SUAPS directors and four wellness instructors (specializing in Yoga, Pilates, and Sophrology). These interviews, with an average duration of 45 min, were carried out either face-to-face or via video conferencing, depending on participants’ schedules, and availability. With participants’ consent, all interviews were audio-recorded and transcribed verbatim for analysis. The semi-structed interview guide, developed by the authors, included questions on various relevant topics, such as the evolution and strategic integration of wellness activities, the impact of COVID-19 on course offerings, resource management, feedback adaptation, as well as facilitators’ qualifications and teaching approaches. This qualitative component allowed us to explore the evolution of wellness programs, the integration of mindfulness practices, and the challenges faced during the COVID-19 pandemic.

### 2.5 Ethical considerations

The study adhered to ethical standards and received approval from the Institutional Review Board of the University of Rouen Normandy (Record 91, Support DPO URN #1870). Informed consent was obtained from all participants prior to their involvement. Anonymity and data confidentiality were upheld throughout the study.

### 2.6 Data analysis

#### 2.6.1 Quantitative data

Quantitative data were analyzed using SAS (Statistical Analysis System). Descriptive statistics, including means, standard deviations, and frequencies, were calculated to describe the sample and key variables. Associations between variables were assessed using inferential statistical methods, such as the Chi-Square test and Fisher's exact test.

#### 2.6.2 Qualitative data

Qualitative data were analyzed using Sphinx software, which facilitated a thorough organization and exploration of key themes, such as stress, student experiences, SUAPS, wellness programs, sports, and COVID-19. Initially, we generated “nuages” (word clouds) to highlight prominent themes, then examined associated verbatim responses to provide context and depth. This approach enabled direct interpretation of the verbatims, revealing recurring patterns and category-specific themes. Through this analysis, we uncovered a shared understanding and collective logic among participants, offering deep insights into the qualitative data.

## 3 Results

### 3.1 Document analysis

This analysis includes 19 studies that focus on the impacts of COVID-19 on the lifestyle and well-being of university students in France. These studies, conducted in various cities such as Rouen, Nîmes, Strasbourg, Lyon, and others, cover diverse aspects of student life during the epidemic. Key areas of focus include the mental health and well-being of university students, particularly the increased stress and anxiety levels experienced during lockdown. Research also examines the pandemic's impact on PA levels, revealing changes in students’ fitness routines. Additionally, some studies investigate changes in dietary behaviors and the prevalence of eating disorders, highlighting the contributing factors to these issues. For the purpose of this research, we will focus on the studies that examined changes in PA due to COVID-19, mental health and well-being, and stress and anxiety responses during lockdown.

#### 3.1.1 Changes in physical activity due to COVID-19

Six of the 19 papers reviewed addressed PA (Table 1), with only two specifically focusing on it. Three studies primarily examined changes in health behaviors (41, 43, 44), while one study investigated factors associated with eating disorders (42). Of the two studies focused on PA, one explored change in PA levels and sedentary behaviors (46), and the other examined the conditions and factors affecting the continuity, cessation, or non-initiation of PA during lockdown (45). The results, that are primarily health-related, indicate that the epidemic has affected students’ PA in several ways. During the lockdowns, there was a significant decrease in both moderate and vigorous PA, and a large number of students stopped participating in sports entirely. However, some students increased their PA during the restrictions, and a considerable portion maintained their exercise routines despite the limitations imposed by the lockdowns. The lockdowns also significantly increased sedentary behavior, raising concerns that these habits might persist even after the restrictions are released.

**Table 1:** Changes in physical activity of French university students due to COVID-19.

Study	Objectives	PA variables	Key findings
Tavolacci et al. (41)	Analyze health behaviors. Evaluate changes and associated factors (+ and -).	Frequency of PA	Significant decrease in moderate PA (from 79.4% to 67.9%; *p* < 0.001) and vigorous PA (from 62.5% to 59.1%; *p* < 0.001) before and during COVID-19. Favorable changes more represented in female students (*p* < 0.001). Negative changes in moderate PA associated with personal infection and feelings of depression anxiety (CESD-8). Positive changes linked to fear of severe COVID-19 contraction. Negative changes in vigorous PA associated with not living with parents during lockdown and CESD-8 score. Positive changes linked to stress from changes in teaching methods.
Tavolacci et al. (42)	Investigate factors associated with eating disorders (EDs).	Frequency of PA	Moderate and vigorous PA are lower in students with EDs compared to those without. The lowest PA levels were observed in students with binge eating disorders. The frequency of PA has decreased in both students with and without EDs during COVID-19.
Patin et al. (43)	Analyze changes in health-related behaviors.	Frequency of PA	Regular moderate PA decreased in May 2020 and May 2021. Occasional moderate PA decreased in May 2020 but returned to pre-COVID-19 levels in May 2021. Regular vigorous PA did not change in May 2020 but decreased in May 2021. Occasional vigorous PA decreased in May 2020 and May 2021.
Matteuci et al. (44)	Compare health-related behaviors of students in France and Italy.	Physical fitness	Before implementation of restrictive measures, 63.3% of French students were physically active. This percentage increased to 72.4% once these measures were in place.
Bouchet-Mayer et al. (45)	Analyze conditions affecting students’ sports practices.	Continuity, interruption, or absence of physical activity during lockdown.	65.1% of students maintained their sports practice despite lockdown restrictions. 23.2% of students stopped their sports practice during lockdown. 11.6% of students who did not have regular sports practice before lockdown did not start any activity during this period.
Goncalves et al. (46)	Describe changes in physical activity levels, sedentary behaviors, and alcohol consumption.	Sedentary behavior and physical activity practices	High PA during the first lockdown, followed by a subsequent decrease during the pandemic. Increased levels of sedentary behavior during both lockdowns, with a tendency for sedentary behaviors to persist over time.

#### 3.1.2 Mental health and well-being

Five studies examined the mental health and well-being of French university students (Table 2), revealing high prevalence of stress, anxiety, depression, and post-traumatic stress disorder (PTSD). Wathelet et al. reported that 42.8% of students experienced at least one mental health symptom, with significant factors being gender, financial instability, and social isolation (49). In 2022, their research indicated a slight decrease in severe stress, anxiety, and depression one-month post lockdown, but an increase in suicidal thoughts (19). They also observed a rise in mental health issues, including PTSD, 15 months after the first lockdown, particularly affecting women, non-binary individuals, and those facing financial difficulties (48). Medical and non-medical health students were found to have a lower risk of adverse mental health outcomes compared to non-health students. Additionally, moderate to severe depressive symptoms were identified in 19.6% of doctoral students, linked to urban confinement and financial difficulties (47, 50). Overall, these studies highlight the significant and long-term impact of the pandemic on students’ mental health, emphasizing the need for targeted interventions.

**Table 2:** Impact of the COVID-19 on French university students’ mental health and well-being.

Study	Objectives	Key findings
Leroy et al. (47)	Risk of mental health problems by type of university studies.	Compared to non-health students, non-medical health students and medical students had a lower risk of presenting at least one adverse mental health outcome. Compared to non-health students, medical students were less at risk of suicidal thoughts, severe self-reported distress, and depression. Non-medical health students were less at risk of severe self-reported distress, stress, depression, and anxiety.
Wathelet et al. (19)	Evolution of Mental Health (1 Month After the 1st Lockdown) and the Impact of Lockdown on Mental Health: Factors Associated With Better or Worse Resilience.	Severe Stress: 24.8% during lockdown, 21.7% one month after lockdown. Anxiety: 27.5% during lockdown, 22.1% one month after lockdown. Depression: 16.1% during lockdown, 13.9% one month after lockdown. Suicidal Thoughts: 11.4% during lockdown, 13.2% one month after lockdown. Factors associated with poor mental health one month after lockdown: female gender, low sense of integration before lockdown, poor quality of social ties during lockdown, history of psychiatric care. Despite a decrease after the first lockdown, severe stress, anxiety, and depression rates remain high, with an increase in suicidal thoughts.
Wathelet et al. (48)	Prevalence of mental health symptoms (15 months after the 1st lockdown) and factors associated with mental health outcomes.	Increased prevalence of stress (20.6%), anxiety (23.7%), depression (15.4%), suicidal thoughts (13.8%), and PTSD (29.8%) at T3 compared to T2. Women and non-binary participants, those without children, living in urban areas, with financial difficulties, chronic conditions, psychiatric history, history of COVID-19, social isolation, and low perceived quality of received information were at risk for all poor outcomes at T3.
Wathelet et al. (49)	Prevalence and associated factors of self-reported mental health symptoms.	1. Prevalence of suicidal thoughts: 11.4%, severe distress: 22.4%, high perceived stress: 24.7%, severe depression: 16.1%, high anxiety: 27.5%. 42.8% of students reported at least one symptom, among which 12.4% consulted a health professional. 2. Risk factors identified include: female or non-binary gender, precariousness (loss of income, poor housing quality), history of psychiatric care, symptoms compatible with COVID-19, social isolation, and poor quality of received information.
Gaudel et al. (50)	Impact of lockdown on the mental health of doctoral students.	89.2% of students rated their health as good or very good. 8.1% presented moderate to severe anxiety symptoms. 19.6% presented moderate to severe depressive symptoms. The prevalence of anxiety disorders was associated with factors such as lack of private outdoor access during lockdown, increased coffee consumption, increased food intake, weight gain, and deteriorated sleep quality. The prevalence of depressive symptoms was associated with factors such as urban confinement, financial difficulties, consulting a health professional, quitting smoking, changes in sleep quantity and quality, and dietary changes.

#### 3.1.3 Stress and anxiety responses during lockdown

Four studies have examined the stress and anxiety of French university students due to COVID-19 (Table 3). These studies indicate that 19.5% of students likely had PTSD, with higher risks among female and non-binary, those living alone, and those experiencing financial difficulties. Factors such as poor social relationships, decrease or loss of income, and inadequate living conditions intensified stress and anxiety levels among students (51). These levels were elevated, especially among students who remained alone in their residences during lockdown (52). Psychological stress was lower among frontline health students; however, their consumption of psychotropics was higher (20). Furthermore, severe felt stress was associated with having a household member hospitalized for COVID-19 (53).

**Table 3:** Impact of the COVID-19 pandemic on stress and anxiety in French university students.

Study	Objectives	Key findings
Wathelet et al. (51)	Report the prevalence rate of probable post-traumatic stress disorder (PTSD) and associated factors among French university students.	The probable PTSD prevalence rate was 19.5%. Associated factors included female or non-binary gender, exposure to a non-COVID-19 related traumatic event, living alone during quarantine, poor quality of social relationships, loss of income, poor housing quality, poor quality of received information, and high exposure to COVID-19. 78.8% of students with probable PTSD considered quarantine potentially traumatic.
Rolland et al. (20)	Evaluate the impact of the first wave of COVID-19 on health students in France, focusing on psychological, educational, and social aspects. Specifically, assess the psychological stress among non-medical health students and the challenges of maintaining educational continuity during university closures due to health regulations.	39% of students reported moderate stress, 21% high levels of stress. Risk factors included: being female, aged 19–21, living alone, and inability to isolate. Frontline students had less psychological stress but better sleep quality and higher consumption of medical and non-medical psychotropics. Nursing and medical students were more stressed and consumed more non-medical psychotropics. The COVID-19 crisis significantly impacted mental health, social life, and education, with disparities by specialty and frontline status.
Husky et al. (52)	Assess levels of stress and anxiety among university students in France during the mandatory COVID-19 lockdown.	Students who did not move during the lockdown were more likely to live alone or with roommates. Those who moved, were more often in rural areas, lived with parents, and had access to outdoor spaces. Most students reported increased anxiety, especially those who stayed in their usual residence. Overall stress was high across the sample, with higher levels for those who did not move, particularly regarding financial and personal health stress. Severe stress was significantly higher among those who stayed in place, especially concerning their health and that of relatives.
Bourion-Bédès et al. (53)	Examine perceived stress levels due to the COVID-19 pandemic and explore associated factors among confined students.	Major risk factors for severe perceived stress included having a household member hospitalized for COVID-19 and being female. Other risk factors included being enrolled in arts, literature, and language programs; postponing a final exam; reduced study time; household conflicts; difficulties isolating; indoor or outdoor noise; lack of direct access to the outdoors. Protective factors included support from friends and family.

### 3.2 Overview of SUAPS offerings and participants in Rouen

**Figure 1:**
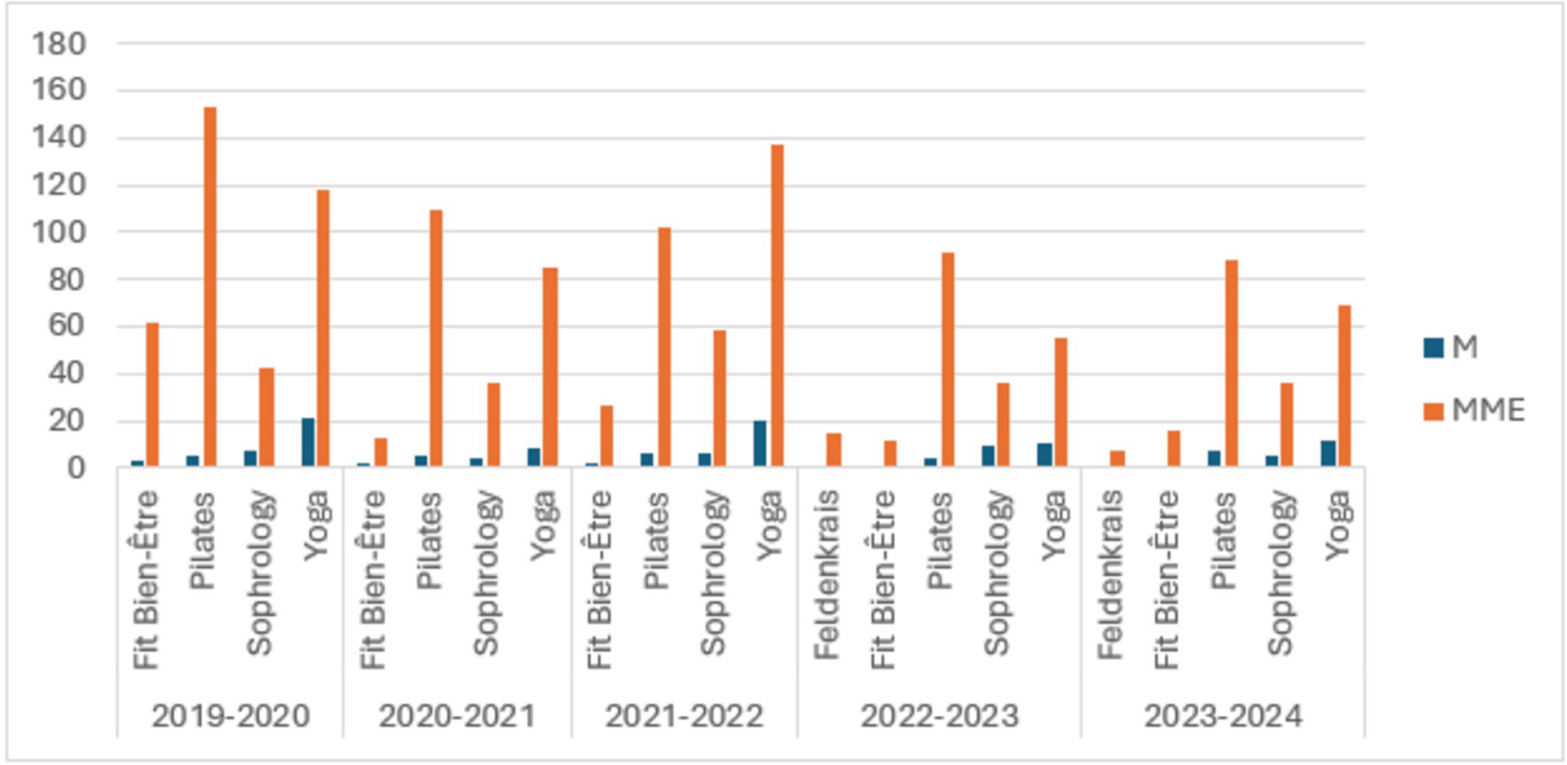
The gender distribution of participation in various wellness activities (Fit bien-Être, pilates, sophrology, yoga, and feldenkrais) at SUAPS over five academic years (2019–2024). Blue bars represent male (M) participation, while orange bars indicate female (MME) participation.

The SUAPS in Rouen offers a diverse array of physical and sports activities for students and staff, including team sports, individual sports, combat sports, wellness programs, and others. The wellness programs aim to improve the emotional and physical health of the university community while also encouraging a balanced and healthy lifestyle. Participation trends in SUAPS’ wellness programs indicate a consistent predominance of female participants in wellness activities, with significantly higher engagement in activities such as yoga, pilates, and sophrology than in male participation. Despite annual fluctuations in overall numbers, the gender distribution remains largely the same (Figure 1).

**Figure 2:**
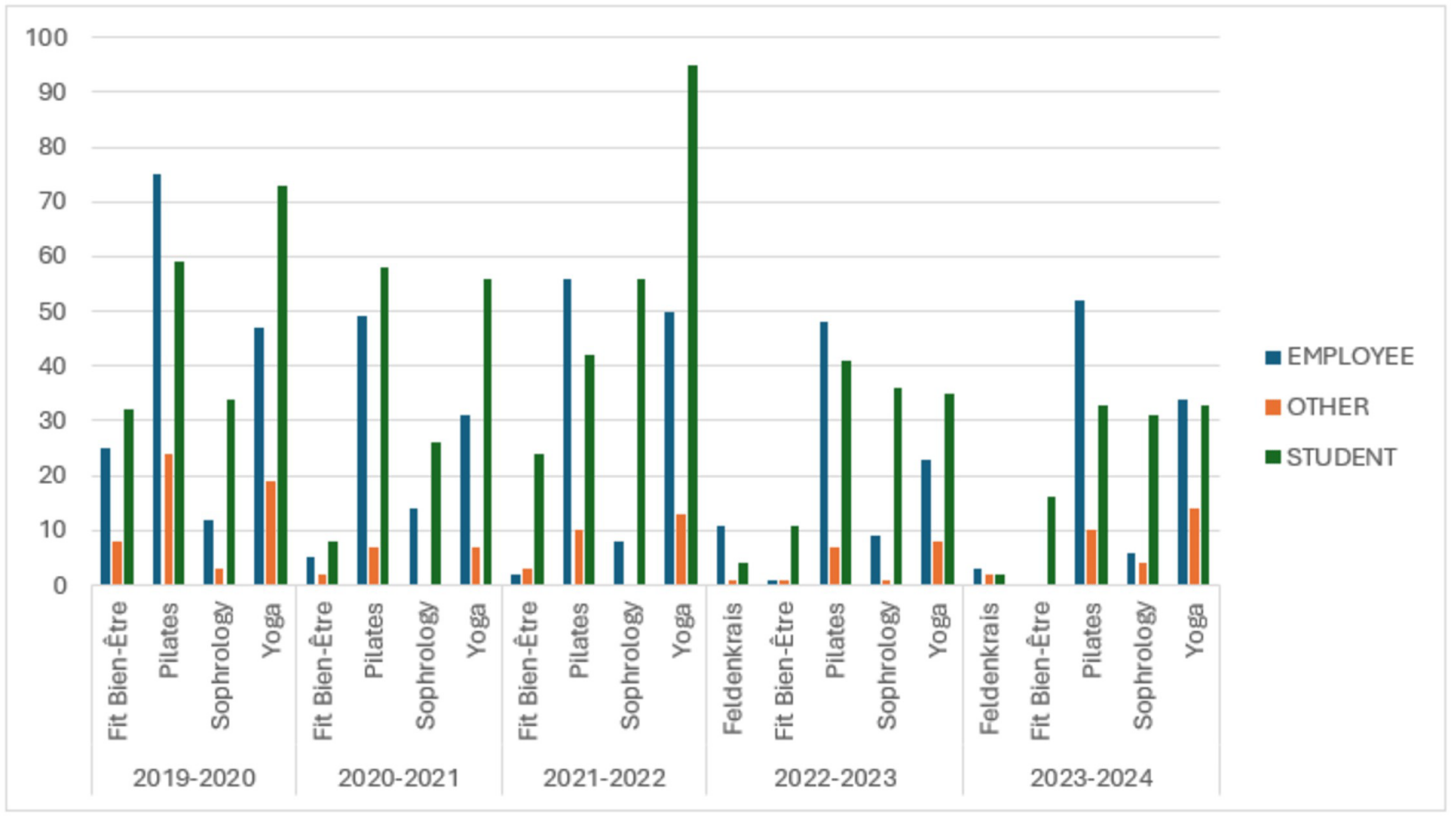
This figure presents the participation of different member types (employee, student, and other) in wellness activities (Fit bien-Être, pilates, sophrology, yoga, and feldenkrais) at SUAPS across five academic years (2019–2024). The blue bars represent employee participation, green bars represent student participation, and orange bars represent participation by other members.

Wellness programs attract both employees and students, with student engagement peaking in the 2021–2022 academic year, when they accounted for 60.5% of total participants, compared to 32.3% of employees. Among the wellness activities, yoga was the most practiced by students, especially in 2021–2022 with 95 student participants, while pilates was the most popular among employees. These insights highlight the broad appeal of the wellness programs at SUAPS, engaging a diverse range of participants from the university community (Figure 2).

**Figure 3:**
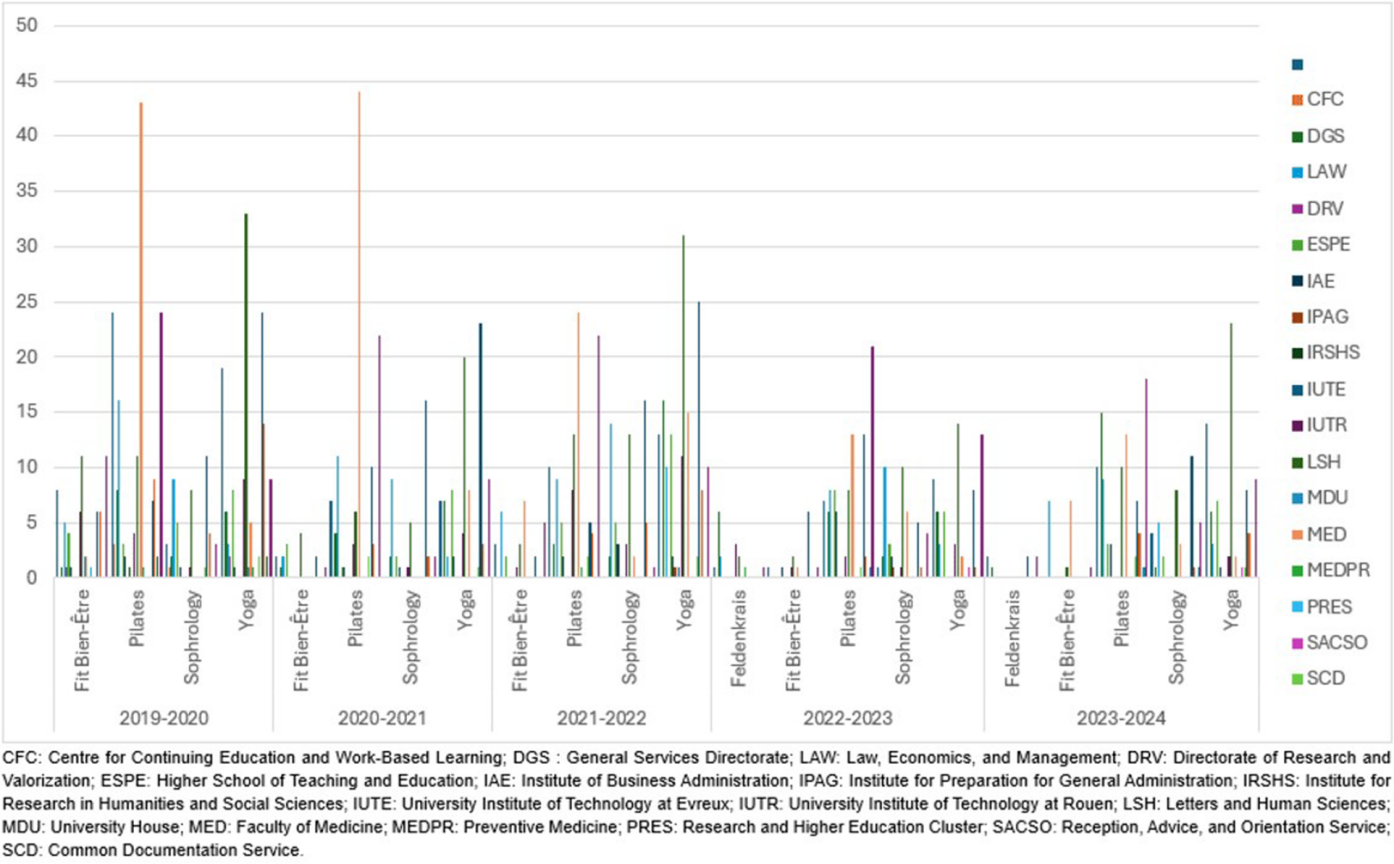
This figure illustrates the participation of various academic divisions in wellness activities (Fit Bien-Être, Pilates, Sophrology, Yoga, and Feldenkrais) at SUAPS across five academic years (2019–2024). Each color represents a distinct academic division, as indicated by the legend on the right.

Additionally, the participation by academic division underscores the widespread engagement across different faculties and administrative sectors. Figure 3 shows significant engagement from a variety of disciplines, including law, medicine, sports science, and others. This distribution emphasizes the inclusive aspect of the wellness programs, which effectively draw participants from all areas of the university, encouraging a holistic approach to health and well-being.

### 3.3 Qualitative study results

This section presents findings from the qualitative study, including insights from both SUAPS directors and well-being activity instructors. Supporting quotes and interpretations are provided in Supplementary Table S1 (ST1) for SUAPS directors and Supplementary Table S2 (ST2) for well-being instructors.

#### 3.3.1 SUAPS directors

##### 3.3.1.1 Theme 1: strategic vision behind the integration of wellness courses at SUAPS

This theme investigates the SUAPS directors’ thoughts on the strategic reasons and motivations for incorporating wellness courses into their offerings. Directors noted that while wellness programs initially targeted university staff in the early 2000s, they gradually expanded to include students, reflecting a shift toward prioritizing student health and well-being. Specific regulatory changes, such as the 2018 decree, and broader health goals accelerated this shift, especially in light of the pandemic. Several initiatives, including those led by Professor Xavier Baguelin, have further reinforced this strategic focus, with the addition of dedicated teaching units for wellness activities. Formerly the director of SUAPS and then the director of the faculty of Sports Science and Physical Education at the University of Rouen during the COVID-19 period, professor Baguelin is now the deputy national director of the French University Sports Federation (FFSU). Under his leadership, SUAPS has also moved from traditional competitive sports to a broader emphasis on wellness, prioritizing student well-being in recent years.

##### 3.3.1.2 Theme 2: structure and pedagogical approach of wellness sessions at SUAPS

This theme investigates the rationale behind the selection of wellness activities at SUAPS, focusing on how these choices are determined by the specific needs and interests of both students and staff. Directors highlighted that wellness activities are chosen based on a variety of factors, including current trends, user demand, and available resources. New activities are frequently introduced based on participant requests or instructor suggestions, with each one being tested to assess its appeal and level of engagement. Feedback from participants is central to refining offerings, ensuring the program remains relevant. Successful implementation of these activities requires careful planning, including securing appropriate facilities and qualified personnel.

##### 3.3.1.3 Theme 3: impact of the COVID-19 pandemic on wellness courses at SUAPS

The COVID-19 pandemic significantly impacted wellness courses at SUAPS, increasing demand and underscoring the importance of wellness for students and staff alike. Directors agreed that the pandemic strengthened wellness practices, with a greater emphasis on developing social connections and well-being when in-person activities resumed. Also, the range of activities remained largely consistent post-pandemic, additional resources were allocated to expand wellness offerings, underscoring their essential role in the SUAPS mission. During lockdowns, activities were adapted for remote access, focusing primarily on staff.

##### 3.3.1.4 Theme 4: future evolution of wellness activities at SUAPS

This theme delves into the projected changes and advances in SUAPS's wellness initiatives throughout the future years. Directors envision expanding the availability of current offerings by adding more time slots, rather than introducing new types of activities, to meet growing demand. They also noted the integration of wellness practices, such as mental and physical preparation, within competitive sports as a way to support athlete health and stress management. Additionally, directors observed a trend in which many people are shifting away from competitive sports and toward wellness-oriented PAs. This trend mirrors a larger change in preferences, with more people selecting activities that enhance well-being, such as Nordic walking.

#### 3.3.2 Well-being activities instructors

##### 3.3.2.1 Theme 1: academic and professional background of SUAPS wellness instructors

This theme delves into the academic and professional backgrounds of SUAPS wellness educators, emphasizing their qualifications, certifications, and specialized training in wellness. The instructors possess diverse backgrounds in various disciplines, enriching SUAPS's wellness program with a broad array of skills. Many have pursued advanced training in both France and abroad, with certifications in areas such as yoga, Pilates, and sophrology. Their expertise enables them to lead high-quality wellness sessions that support the mental and physical well-being of students and staff, customized to meet their unique needs.

##### 3.3.2.2 Theme 2: structure and pedagogical approach of wellness sessions at SUAPS

This theme explores the organization and teaching methods of SUAPS wellness sessions, focusing on a variety of exercises, body awareness, and the interactive, adaptive nature of the sessions. Instructors tailor each session to meet varying skill levels. SUAPS’ wellness sessions include a variety of exercises customized to the needs and levels of the participants. These exercises incorporate components of yoga, breathing methods, balance, relaxation and visualization. Breathing exercises, particularly pranayama, are integral to fostering conscious breathing and overall well-being. The sessions follow a progressive approach, starting with basic exercises and advancing to more complex movements that engage multiple muscles and joints.

##### 3.3.2.3 Theme 3: impact of the COVID-19 pandemic

This theme examines the influence of the COVID-19 pandemic on student participation in SUAPS wellness courses, focusing on changes in attendance, commitment, and activity preferences. The pandemic did not significantly impact student interest in wellness classes; most students continued their pre-existing levels of involvement primarily to fulfill mandatory wellness credits, with no notable increase in interest due to the pandemic. However, a slight increase in young people's engagement in Pilates classes was observed, indicating a growing preference for this specific activity. Among adults, there was a remarkable consistency and strong commitment to continue Pilates during the epidemic, demonstrating a difference in how distinct groups responded to health activities during this time.

##### 3.3.2.4 Theme 4: challenges in youth engagement and strategies for overcoming them

This theme addresses the challenges instructors encounter in engaging young participants in wellness courses at SUAPS and the strategies employed to enhance commitment and focus. Key challenges include maintaining student engagement over time, managing attention during sessions amid distractions like cell phones, and dispelling stereotypes associated with wellness activities such as yoga and Pilates. To improve concentration, instructors encourage students to set aside their phones and implement customized programs that cater to varying skill levels. Addressing stereotypes and creating a positive learning environment are essential for encouraging ongoing participation and openness toward wellness practices. Programs are designed to support diverse abilities, allowing beginners to progressively build their skills while enabling experienced participants to continue advancing without feeling limited.

### 3.4 Quantitative study results

#### 3.4.1 Sociodemographic characteristics of the population

The study consisted of 218 participants, with a mean age of 33 years (SD = 15.35). Females comprised 77.98% of the group. Most participants were from Université de Rouen Normandie (70.64%), while 29.36% were from Université de Paris Cité. The Science and Technology faculty had significant representation at 20.64%, followed by Law, Economics, and Management at 9.63%. Regarding employment status, 44.50% of participants were employed, while 55.50% were unemployed (Table 4). In the youth subgroup (*N* = 134), 76.12% were female, and 72.39% were associated with the Université de Rouen Normandie. The humanities and Social Sciences department was the most represented, accounting for 27.07% of the subgroup. Additionally, 66.42% of the youth were employed, with the majority (70%) pursuing or had completed a bachelor's degree (ST3).

**Table 4:** Sociodemographic characteristics of the study group (*N* = 218).

	*N*	%
Age ^a^		
18–35 years	134	62.91
35–59 years	70	32.86
≥ 60 years	9	4.22
Gender		
Female	170	77.98
Male	48	22.02
University		
Université de Paris Cité	64	29.36
Université de Rouen Normandie	154	70.64
Faculty		
Law, economics, and management	21	9.63
Arts and humanities	27	12.39
Health sciences	21	9.63
Human and social sciences	23	10.55
Science and technology	45	20.64
Science and techniques of physical and sport activities (STAPS)	12	5.96
Other	68	31.19
Department ^a^		
Business and economics	21	9.68
Languages and literature	16	7.37
Health and medicine	22	10.14
Science and engineering	49	22.58
Humanities and social sciences	38	17.51
Science and techniques of physical and sport activities (STAPS)	11	5.07
Technology and computer sciences	3	1.38
Other	57	26.27
Employment status		
Employed	97	44.50
Unemployed	121	55.50

^a^ Missing values.

#### 3.4.2 Physical activity levels and sports practice patterns

As presented in Table 5, 48.16% of the total population and 50% of the youth engaged in high levels of PA. Moderate PA levels were reported by 42.20% of the overall population and 38.81% of the youth, while low activity levels were less common. Outdoor activities were the most popular sport among all participants (18.52%), followed by fitness and strength training (15.29%) and athletic sports (12.96%). In the youth group, fitness and strength training were the most preferred (18.18%), followed by team sports (17.42%) and athletic sports (14.39%). Session duration varied, with most participants engaging for 30 min–1.5 h. In the total population, 39.45% reported sessions of 30 min–1 h, and 37.16% for 1–1.5 h. The youth group had a similar pattern, with 41.04% reporting 1–1.5-hour sessions. Participants’ primary goals were improving physical condition (86.24% overall, 79.85% youth) and mental well-being (63.76% overall, 61.95% youth). Most participants practiced sports outside a club (74.31% overall, 73.88% youth) and preferred indoor activities (74.31% overall, 80.60% youth), though many also engaged in outdoor sports (43.12% overall, 38.81% youth).

**Table 5:** Physical activity levels and sports practice patterns.

	Total (*n* = 218)				Youth (*n* = 134)			
	*n*		%		*n*		%	
Physical activity level								
Low	21		9.63		15		11.19	
Moderate	92		42.20		52		38.81	
High	105		48.16		67		50.00	
Preferred sport^a^								
Dance	20		9.26		11		8.33	
Fitness and strength training	33		15.29		24		18.18	
Aquatic sports	17		7.87		10		7.58	
Athletic sports	28		12.96		19		14.39	
Team sports	25		11.57		23		17.42	
Combat sports	12		5.56		9		6.82	
Outdoor sports	40		18.52		13		9.85	
Racquet sports	11		5.09		8		6.06	
Cardio sports	5		2.31		3		2.27	
Yoga	9		4.17		4		3.03	
Pilates	16		7.41		8		6.06	
Session duration								
Less than 30 min	12		5.50		7		5.22	
Between 30 min and 1h	86		39.45		46		34.33	
Between 1 h and 1.5h	81		37.16		55		41.04	
More than 1.5 h	39		17.89		26		19.40	
Goals of sports practice	Yes	%	No	%	Yes	%	No	%
Physical condition	188	86.24	30	13.6	107	79.85	27	20.15
Weight loss	61	27.98	157	72.02	38	28.36	96	71.64
Muscle gain	40	18.35	178	81.65	27	20.14	107	79.86
Flexibility improvement	36	16.51	182	83.49	18	13.43	116	86.57
Mental wellbeing enhancement	139	63.76	79	36.24	83	61.95	51	38.05
Socialization	42	19.27	176	80.73	28	20.90	106	79.10
Performance	52	23.85	166	76.15	48	35.82	86	64.18
Sport practice modalities								
In a club	85	38.99	133	61.01	51	38.06	83	61.94
Outside a club	162	74.31	56	25.69	99	73.88	35	26.12
Indoors	162	74.31	56	25.69	108	80.60	26	19.40
Outdoors	94	43.12	124	56.88	52	38.81	82	61.19

h, hours; min, minutes.

^a^ Missing values.

#### 3.4.3 Wellness and mindfulness practices

Overall, Participants reported diverse engagement in wellness activities, with many practicing for less than 6 months or over 5 years. The wellness and mindfulness offerings in SUAPS are non-competitive, focusing on enhancing student's overall well-being and health. Among these activities, Fitness well-being (30.28%) and Pilates (29.36%) were the most popular activities overall, with similar trends in the youth group (32.84% for fitness and 19.4% for Pilates). Yoga was also a significant activity (21.10% overall, 17.91% youth). The main reasons for engaging in wellness activities included improving physical condition (77.98% overall, 68.66% youth), mental health (51.83% overall, 60.45% youth), and stress management (47.25% overall, 48.51% youth). In addition, 36.70% of the total population and 38.81% of the youth regularly practice wellness and mindfulness activities outside SUAPS, while others did so occasionally or not at all. These activities were most often practiced less frequently, with 46.88% of the total group and 51.76% of the youth reporting infrequent engagement. Daily practice was reported by 15.63% overall and 15.29% of the youth. Most participants preferred short mindfulness sessions, with 54.31% of the total group and 51.32% of the youth practicing for less than 10 min (Table 6).

**Table 6:** Wellness and mindfulness practices.

	Total (*n* = 218)		Youth (*n* = 134)	
	*n*	%	*n*	%
Wellness activity				
Sophrology	16	7.34	10	7.46
Yoga	46	21.10	24	17.91
Fitness well-being	66	30.28	44	32.84
Feldenkrais	3	1.38	2	1.49
Pilates	64	29.36	26	19.4
Meditation	22	10.09	13	9.70
Other	17	7.79	9	6.71
Reasons for wellness activities				
Physical condition	170	77.98	92	68.66
Mental health	113	51.83	81	60.45
Social connections	25	11.47	19	14.18
Stress management	103	47.25	65	48.51
Other	10	4.58	2	1.49
Practicing wellness outside SUAPS				
No	76	34.86	42	31.34
Yes, occasionally	62	28.44	40	29.85
Yes, regularly	80	36.70	52	38.81
Wellness Duration				
Less than 6 months	45	20.64	44	32.84
6 months–1 year	47	21.56	35	26.12
1–3 years	50	22.94	29	21.64
3–5 years	21	9.63	6	4.48
More than 5 years	55	25.23	20	14.93
Mindfulness Frequency ^a^	*n* = 128		*n* = 85	
Daily	20	15.63	13	15.29
Several times a week	25	19.53	15	17.65
Weekly	23	17.97	13	15.29
Less frequently	60	46.88	44	51.76
Mindfulness session duration ^a^	*n* = 116		*n* = 76	
Less than 10 min	63	54.31	39	51.32
Between 10 and 20 min	21	18.10	14	18.42
Between 20 and 30 min	13	11.21	10	13.16
Between 30 min and 1h	9	7.76	7	9.21
More than 1h	10	8.62	6	7.89

H, hours; min, minutes.

^a^ Missing values.

#### 3.4.4 COVID-19 period: sports participation and correlating factors

66.51% of the total group and 58.96% of the youth reported contracting COVID-19 at least once. Despite this, many continued engaging in sports, though activity levels varied: group and 42.54% of the youth decreased their activity, 28.44% overall and 33.58% of the youth increased it. The lockdown also encouraged participants to explore new sports, with fitness and strength training being the most popular, especially among the youth (70.68%). Other newly adopted activities included outdoor sports (22.72% overall, 18.96% youth) and yoga and Pilates, (19.31% and 15.90%, respectively). Post-lockdown, 58.72% of participants resumed their sports activities, though this was less common among the youth (49.25%). However, 25.69% of the total group and 29.10% of the youth did not return to their previous sports routines, primarily due to lack of motivation (24.31% overall, 29.85% youth), changing priorities (22.48% overall, 22.39% youth), and health concerns (6.88% overall, 7.46% youth) (Table 7).

**Table 7:** Impact of lockdown on sport practice and activity adaptations Among participants.

	Total (*n* = 218)		Youth (*n* = 134)	
	*n*	%	*n*	%
COVID positive				
0	73	33.49	55	41.04
1	75	34.40	41	30.60
2	53	24.31	31	23.13
3 or more	17	7.80	7	5.23
Sport practice during lockdown				
Yes	118	54.13	72	53.73
No	100	45.87	62	46.27
Lockdown exercise adaptations				
Individual practice	124	56.88	83	61.94
Outdoor exercises	70	32.11	37	27.61
Other	5	2.29	3	1.37
Sport level during lockdown				
Increased	62	28.44	45	33.58
Decreased	106	48.62	57	42.54
Unchanged	50	22.94	32	23.88
New sport during lockdown				
Yes	86	39.45	58	43.28
No	132	60.55	76	56.72
New activities during Lockdown ^a^	*n* = 88		*n* = 58	
Dance	5	5.68	4	6.89
Fitness and strength	53	60.22	41	70.68
Pilates	14	15.90	8	13.79
Yoga	17	19.31	11	18.96
Meditation	2	2.27	2	3.44
Outdoor sports	20	22.72	11	18.96
Cardio sports	17	19.31	10	17.24
Team sports	3	3.40	2	3.44
Athletic sports	15	17.04	10	17.24
Combat sports	2	2.27	2	3.44
Resuming sport post-lockdown				
Yes	128	58.72	66	49.25
No	56	25.69	39	29.10
Not applicable	34	15.60	29	21.64
Reasons not resuming sport				
Lack of motivation	53	24.31	40	29.85
Health concerns	15	6.88	10	7.46
Priority changes	49	22.48	30	22.39
Other	14	6.42	8	5.97

^a^ Missing values.

The study found significant correlations between COVID-19 positivity and several demographic factors: age (*p* = 0.0143), with adults and seniors more likely to contract the virus multiple times compared to youth, indicating higher vulnerability in older age groups (ST4); education level (*p* = 0.0000), with infection rates varying among individuals with higher education, such as PhD candidates and HDR holders, suggesting differing exposure risks at different educational stages (ST5); employment status (*p* = 0.0005), as employed individuals had higher infection rates, likely due to increased exposure in work settings (ST4); and faculty affiliation (*p* < 0.0001), with infection rates varying across different academic disciplines, indicating differing exposure risks (ST6). However, no significant correlation was found between PA levels (*p* = 0.7702) or engaging in sports during the lockdown (*p* = 0.9585) and COVID-19 positivity, suggesting that these factors did not influence the likelihood of infection (ST7 & ST8). Additionally, the analysis of sports practice and wellness activities reveals a significant association, with youths involved in sports being more likely to engage regularly in wellness activities outside SUAPS (*p* < 0.0001) (ST9). Moreover, individuals who participate in sports tend to engage in wellness activities for longer periods compared to those who don't, demonstrating a strong connection between sports participation and long-term commitment to wellness practices (*p* < 0.0005) (ST10).

#### 3.4.5 Stress levels and wellness: key insights and correlations

The overall population had a mean PSS score of 18.86 (SD = 6.81), slightly lower than the youth subgroup's mean of 19.17 (SD = 6.75). Median scores were similar, with 19.00 for the total group and 18.00 for the youth. Moderate stress was the most prevalent, affecting 64.68% of the total population and 64.18% of the youth group, while low and high stress were reported by around 23% and 12% in both groups, respectively (Table 8). These results suggest similar stress patterns in both groups, with moderate stress being the most prevalent.

**Table 8:** Stress levels in the study group and youth participants.

	Total (*n* = 218)		Youth (*n* = 134)	
PSS score				
Mean	18.86		19.17	
SD	6.81		6.75	
Median	19.00		18.00	
Stress levels	*n*	%	*n*	%
Low	50	22.94	31	23.13
Moderate	141	64.68	86	64.18
High	27	12.39	17	12.69

The relationship between wellness activities, the reasons for engaging in them, and participant stress levels, as shown in Tables 9, 10, revealed no direct link between specific wellness activity and stress levels, moderate stress was consistently the most commonly reported across all activities. However, motivations for participating in wellness activities, particularly for mental health and stress management, showed significant correlations with stress levels. Among the engaging for mental health reasons, 64.68% of the study group and 64.18% of the youth group reported moderate stress, with significant *p*-values of 0.0000 and 0.0022, respectively. Similarly, stress management motivations also showed significant correlations with stress levels (*p* = 0.0024 in the study group and *p* = 0.0160 in the youth group, indicating that higher stress levels may drive individuals to seek wellness activities for mental health support.

**Table 9:** Correlations between wellness activities and stress levels in the study and youth groups.

Stress level	Total count		Sophrology		Yoga		Fitness well-being		Feldenkrais		Pilates		Meditation		Other	
	*n*	%	*N*	%	*N*	%	*N*	%	*N*	%	*N*	%	*N*	%	*N*	%
Study group																
Total	218	100.00	16	100.00	46	100.00	66	100.00	3	100.00	64	100.00	22	100.00	17	100.00
Low	50	22.94	1	6.25	9	19.57	17	25.76			15	23.44	2	9.09	3	17.65
Moderate	141	64.68	12	75.00	30	65.22	42	63.64	3	100.00	41	64.06	15	68.18	12	70.59
High	27	12.39	3	18.75	7	15.22	7	10.61			8	12.50	5	22.73	2	11.76
*P* -value			0.1874		0.7157		0.7458		0.7102		0.9919		0.1222		0.2343	
Youth																
Total	134	100.00	10	100	24	100.00	44	100	2	100.00	26	100.00	13	100.00	9	100.00
Low	31	23.13	1	10.00	4	16.67	9	20.45			4	15.38	2	15.38	1	11.11
Moderate	86	64.18	7	70.00	17	70.83	30	68.18	2	100.00	17	65.38	9	69.23	7	77.78
High	17	12.69	2	20.00	3	12.50	5	11.36			5	19.23	2	15.38	1	11.11
*P* -value			0.4929		0.6938		0.7956		1.0000		0.3803		0.8291		0.6111	

Statistical analyses were performed using Fisher's test and Chi-Square test, with a *p*-value <0.05 considered statistically significant.

**Table 10:** Correlations between wellness practice reasons and stress levels in the study and youth groups.

Stress level	Total count		Physical condition		Mental health		Social connections		Stress management		Other	
	*n*	%	*N*	%	*N*	%	*N*	%	*N*	%	*N*	%
Study group												
Total	218	100.00	170	100.00	113	100.00	25	100.00	103	100.00	10	100.00
Low	50	22.94	37	21.76	14	12.39	5	20.00	14	13.59	6	60.00
Moderate	141	64.68	111	65.29	76	67.26	17	68.00	71	68.93	2	20.00
High	27	12.39	22	12.94	23	20.35	3	12.00	18	17.48	2	20.00
*P* -value			0.7049		0.0000*		0.9242		0.0024*		0.5810	
Youth												
Total	134	100.00	92	100.00	81	100.00	19	100.00	65	100.00	2	100.00
Low	31	23.13	21	22.83	11	13.58	5	26.32	9	13.85	2	100.00
Moderate	86	64.18	59	64.13	56	69.14	13	68.42	44	67.69		
High	17	12.69	12	13.04	14	17.28	1	5.26	12	18.46		
*P* -value			0.9794		0.0022*		0.6645		0.0160*			

* Statistical analyses were performed using Fisher's test and Chi-Square test, with a *p*-value <0.05 considered statistically significant.

Further analysis reinforced the connection between wellness practices and stress levels. A significant correlation was found between wellness activities outside SUAPS and stress levels, with regular participants reporting lower stress (35%) than occasional (12.9%) and non-practitioners (18.42%), supported by a *p*-value of 0.0193 (ST11). However, no significant correlations were found between stress levels and the duration of wellness practice (*p* = 0.3660) (ST12) or the length of individual wellness sessions (*p* = 0.3463) (ST13). On other hand, COVID-19 positivity did not significantly affect stress levels in either group (Table 11). However, within the youth group, regular sport practice was significantly associated with lower stress levels (*p* = 0.0400) (Table 12). These findings highlight the crucial role of consistent engagement in wellness and sports activities for effective stress management, particularly among youth.

**Table 11:** Distribution of stress levels by COVID-19 Status.

COVID positive	Total count		Low		Moderate		High		*P*-value
	*n*	%	*N*	%	*N*	%	*N*	%	
Study group									
Total	218	100.00	50	100.00	141	100.00	27	100.00	0.5695
0	73	33.49	18	36.00	49	34.75	6	22.22	0.5695
1	75	34.40	18	36.00	44	31.21	13	48.15	
2	53	24.31	9	18.00	38	26.95	6	22.22	
3 or more	17	7.80	5	10.00	10	7.09	2	7.41	
Youth									
Total	134	100.00	17	100.00	31	100.00	86	100.00	0.9638
0	55	41.04	6	35.29	14	45.16	35	40.70	0.9638
1	41	30.60	5	29.41	8	25.81	28	32.56	
2	31	23.13	5	29.41	7	22.58	19	22.09	
3 or more	7	5.22	1	5.88	2	6.45	4	4.65	

Statistical analyses were performed using Fisher's test and Chi-Square test, with a *p*-value <0.05 considered statistically significant.

**Table 12:** Correlation between sport practice and stress levels in study and youth groups.

Stress level	Total count		No		Yes		Chi-square test: *P*-value
	*n*	%	*N*	%	*N*	%	
Study group							
Total	218	100.00	32	100.00	186	100.00	0.0542
Low	50	22.94	3	9.38	47	25.27	
Moderate	141	64.68	22	68.75	119	63.98	
High	27	12.39	7	21.88	20	10.75	0.0542
Youth							
Total	134	100.00	23	100.00	111	100.00	0.0400*
Low	31	23.13	2	8.70	29	26.13	
Moderate	86	64.18	15	65.22	71	63.96	
High	17	12.69	6	26.09	11	9.91	0.0400*

* *P*-value <0.05 indicates statistical significance.

## 4 Discussion

This study investigates the impact of wellness and mindfulness practices on stress levels and their influence on PA engagement among youths, particularly in the wake of the COVID-19 pandemic. Our review emphasizes the significant impact of COVID-19 and lockdowns on university students in France, who often lack the coping mechanisms of older adults. Key stressors include social isolation, financial instability, and academic disruptions. According to Husky et al., one of the main causes of the elevated stress levels among students living alone during lockdowns is social isolation (52). Likewise, Wathelet et al. identified financial instability as a significant stressor, further contributing to the heightened vulnerability of youth (48). These findings align with our data, which show that the youth subgroup reported slightly elevated stress levels compared to the general population. Our 2024 results further reveal that the pandemic's effects on youth stress remain significant even three years after the lockdowns ended. Young people faced an intense layering of stressors: beyond being perceived as more contagious and often asymptomatic, they feared transmitting the virus to elderly family members, leading to guilt and anxiety (54, 55). The closures of activities, forced isolation, repeated testing, increased screen time, loss of student jobs, limited transportation, and social restrictions profoundly affected their daily lives. Many also experienced poor dietary habits and were impacted by the economic crisis, further exacerbating stress (22, 23, 25, 56). In our study, 64.18% of youth participants reported moderate stress levels, and 12% experienced high stress, indicating that the psychological strain from the pandemic has not fully receded. This accumulation of stressors led to a rise in stress levels while simultaneously reducing access to regular PAs and social interactions that could have provided a protective effect (57). Youth people remain particularly vulnerable to the pandemic's long-term impact on mental health, underscoring the need for sustained interventions and support.

The study also revealed that COVID-19 positivity was strongly linked to demographic parameters such age, education, employment, and faculty affiliation. Older adults and seniors were more vulnerable to recurring infections than younger individuals, highlighting age-related risks. Higher infection rates among PhD candidates and employed participants suggest that individuals with in-person responsibilities faced greater exposure (58, 59). In addition, the differences in infection rates among academic faculties indicate that disciplines involving more in person interactions had a higher exposure risk. These patterns highlight the complex link between stress, well-being, and exposure to COVID-19, especially for students managing both mental health challenges and elevated infection risk.

Despite lockdowns and restrictions, 33.58% of the young participants in our study reported an increase in their PA levels, and 23.88% managed to maintain their routines. This demonstrates the resilience and adaptability of youth, who likely experimented with new activities and turned to alternative techniques, such as home workouts, online fitness programs, or outside exercises allowed during lockdowns. These results align with Bouchet-Mayer et al., who found that 65.1% of students were able to maintain or even improve their PA routines during the pandemic (45). This resilience can be linked to the flexibility and adaptability often seen in younger individuals, enabling them to stay active despite restrictive conditions (60). Additionally, PA may have served as a vital coping mechanism, offering a sense of normalcy and control amidst the stress and uncertainty brought on by the pandemic. Besides, 60.2% of the total population and 55.15% of the youth who explored new activities during lockdown engaged in wellness and mindfulness practices such as yoga, pilates, and Nordic walking. These observations align with those made by SUAPS directors, who noted a shift from competitive sports to wellness-focused activities, reflecting a societal move towards prioritizing mental and physical well-being during times of crisis.

While many participants reported an increase in sports practice, 48.62% of the overall population and 42.54% of youth reported a decrease in PA during lockdowns. Additionally, 25.69% of adults and 29.10% of youth did not resume their sports practices after lockdowns. These findings are consistent with the studies conducted by Tavolacci et al., Patin et al., and Bouchet-Mayer et al. in France as well as international studies by Park AH et al. and Do B et al., which observed a global decline in PA during COVID-19 lockdowns (26, 41, 43, 45, 61). Several factors contributed to the decline in PA during lockdowns. The closure of gyms, sports facilities, and restrictions on outdoor movement severely limited opportunities for exercise, particularly for those dependent on structured environments. The disruption of daily routines, such as commuting or team sports, led to increased sedentary behavior as many transitioned to remote work or study. Heightened stress, uncertainty, and isolation further undermined motivation, making it challenging for many to prioritize PA. This decrease in activity also contributed to higher stress levels, as PA naturally helps mitigate stress by releasing endorphins and reducing cortisol (62, 63). Our study found that regular sports practice was associated with lower stress, particularly among youth, where a statistically significant correlation (*p* = 0.0400) was found between maintaining sports activity and reduced stress. These findings reinforce the role of PA as an essential strategy for managing stress, particularly during challenging periods such as the COVID-19 pandemic.

The analysis found no statistically significant correlation between specific wellness activities and stress reduction, with all activities yielding *p*-values above 0.05. This suggests that the type of activity may not be the primary factor in stress reduction; rather individual differences such as personal preferences and engagement levels, along with external stressors and overall mental health, are likely important influences. These results are consistent with earlier research showing that that the effectiveness of wellness activities depends more on personal engagement and practice quality than on the specific type of intervention (64–66). Additionally, while the type of wellness activity alone did not affect stress levels, our study revealed a significant link between participants’ reasons for engaging in these practices and their stress outcomes. Those who pursued wellness activities mainly for mental health reasons consistently reported moderate stress levels. Especially when it comes to mental health and stress management, participants who engaged in wellness practices primarily for mental health reasons consistently reported moderate stress levels, highlighting the importance of personal motivation and intent in the effectiveness of wellness practices.

Beyond the type of activity, our study underscores the importance of consistency in wellness and mindfulness practices for effective stress management. Regular engagement in wellness activities was associated with significantly lower stress levels compared to occasional participation or no participation. These findings are supported by previous reviews, which highlight that regular practice is key to effective stress management (65–67). This underscores the importance of regular engagement in wellness activities over the specific type of practice, as it helps develop a reliable method for ongoing stress management. Additionally, our results show that the duration or intensity of individual wellness sessions did not significantly impact stress reduction. Feedback from SUAPS instructors further supports this perspective, noting that shorter wellness and mindfulness sessions were particularly beneficial during the pandemic when time was limited. They emphasize that the intensity of sessions is not critical for obtaining the benefits of wellness and mindfulness programs, which are inherently personal and adaptable to individual needs.

Finally, our findings indicate a substantial link between sports practice and involvement in wellness activities, with youths involved in sports demonstrating higher rates of regular participation and long-term commitment. This suggests that sports practice positively influences participation in wellness activities. Incorporating mindfulness practices, such as meditation and yoga, could further strengthen this connection. Integrating mindfulness with PA creates a well-rounded health approach, fostering ongoing involvement and enhancing the enjoyment and ease of maintaining a sports routine.

In light of these findings, it is clear that somatic practices and body ecology have become essential strategies for managing stress and adapting to new health challenges, such as those brought on by the COVID-19 pandemic (68). Today's youth, coming of age in the Anthropocene, a period shaped by profound human impact on the planet, are confronting a wide range of complex stressors, from climate change and pollution to economic instability and evolving social landscapes. Consequently, young individuals need strategies to navigate the unique and intensified challenges of our time that are exacerbated by these pressures (69, 70). Studies conducted at the Universities of Rouen Normandy and Paris Cité provides strong examples of successful collaborations between research laboratories and adapted physical activity (APA) programs, as well as the certification of Sports and Health Centers.

In 2018, ASRUC Santé was launched in Rouen, at the forefront of implementing the Fourneyron Law [named after Rouen's former mayor and Minister of Sports]. As part of the Rouen University Club Sports Association (*Association Sportive Rouen Université Club*, ASRUC), it became the first multi-sport club in Normandy to establish a dedicated sports and health section. ASRUC Santé's mission is to offer tailored PAs that address the specific health needs of its members under the decree promoting “exercise on prescription,” aiming to enhance participants’ overall health, as well as their physical and psychological well-being. All sessions are led by certified educators in APA for health. To optimize care for its members, ASRUC Santé collaborates with a dietitian-nutritionist and a sophrologist. A variety of free fitness activities are offered to students, including mobility and stretching, mobility and strength training, Nordic walking, and health cross ‘fit (71).

Additionally, following various studies conducted at Paris Cité University, a new methodology was developed to track students during the post-COVID period. The I3SP is actively involved in the experimental implementation of the university sports and health program, designed to promote “exercise on prescription” for eligible students while addressing the growing concerns of physical inactivity and sedentary behavior within the student population. The lab works closely with the Student Health Service (*Service de Santé Étudiante*, SSE) and the university's Sports and Physical Activities Department (*Service Universitaire des Activités Physiques et Sportives*, SUAPS). This three-year project is co-financed by the Regional Health Agency (*Agence Régionale de Santé*, ARS), the Regional Center for University and School Works (*Centre Régional des Œuvres Universitaires et Scolaires*, CROUS), and the three universities (Paris 1, Paris 3, and Paris Cité) (72).

This study has certain limitations, including its focus on participants primarily from the University of Rouen, which limits the generalizability of the findings to all French university students. The uneven distribution between Rouen and Paris participants further restricts regional comparisons, especially given the smaller sample size from Paris. This imbalance, combined with the relatively small sample size from Paris, restricts our capacity to draw robust comparisons between students in different academic and geographic contexts. Additionally, using both face-to-face and videoconference interviews may have influenced participant interactions, with potential differences in non-verbal cues and formality affecting the depth of responses. The timing of our study, coinciding with the Olympic year in France, also posed challenges, as some participants were involved in Olympic preparations, which further limited their availability. Despite its limitations, this study has several key strengths. The multi-method approach, which integrates a literature review, surveys, and interviews, provides a thorough understanding of COVID-19's impact on student well-being and PA. Emphasizing wellness and mindfulness practices, an area that has received less attention in student stress management, offers important insights for future interventions. The diverse participant pool, spanning different ages and academic levels, enriches the findings. Additionally, the study adhered to strong ethical standards and employed rigorous statistical and qualitative methods, enhancing the reliability of the results. In summary, this research offers valuable insights into enhancing student well-being both during the pandemic and in the future.

## 5 Conclusion

In conclusion, this study emphasizes the long-term impact of the COVID-19 pandemic on youth stress levels and highlights the crucial role of wellness, mindfulness activities, and PA in effectively managing this stress. Despite the challenges, youth showed remarkable resilience by increasing their engagement in both PA and wellness practices during the pandemic. These findings suggest that incorporating mindfulness and wellness activities into educational settings may enhance students’ mental resilience and overall well-being.

In France, a promising approach is the development and expansion of “Maisons Sport-Santé” within universities. These centers promote PA, healthy dietary habits, and overall well-being through personalized support and guidance, to improve students’ quality of life. Currently available in select universities, Maisons Sport-Santé aim to make healthy lifestyles more attainable with tailored resources, programs, and personalized guidance. Expanding these platforms to all French universities would allow more students to benefit from this structured health support. To maximize their reach, these wellness centers should collaborate with SUAPS, which already manages PA programs, to integrate and promote a range of wellness and mindfulness activities as core aspects of student life. In turn, SUAPS could expand their offerings to include diverse mindfulness and wellness options.

Moreover, these findings point to the importance of further research on how the Anthropocene affects youth stress and health behaviors. Building on existing studies, future research could examine links between human-driven environmental changes, somatic practices, body ecology, and deep ecology, uncovering innovative ways to foster resilience through mindfulness. Such interdisciplinary perspectives provide youth with tools to address immediate stress and build lasting mental resilience. Establishing educational environments that incorporate these practices is essential to preparing young people for a balanced, resilient future.

## Data Availability

The original contributions presented in the study are included in the article/Supplementary Material, further inquiries can be directed to the corresponding authors.

## References

[1] de Greeff JW Bosker RJ Oosterlaan J Visscher C Hartman E. Effects of physical activity on executive functions, attention and academic performance in preadolescent children: a meta-analysis. J Sci Med Sport. (2018) 21(5):501–7. 10.1016/j.jsams.2017.09.595 29054748

[2] Ludyga S Gerber M Brand S Pühse U Colledge F. Effects of aerobic exercise on cognitive performance among young adults in a higher education setting. Res Q Exerc Sport. (2018) 89(2):164–72. 10.1080/02701367.2018.1438575 29513092

[3] Soorgi S Godarzi Y Shabib Asl N Ghorbani M. The effectiveness of executive function training on students’ academic vitality and academic performance. Pajouhan Sci J. (2021) 19(5):43–50. 10.61186/psj.19.5.43

[4] Srinivas NS Vimalan V Padmanabhan P Gulyás B. An overview on cognitive function enhancement through physical exercises. Brain Sci. (2021) 11(10):1289. 10.3390/brainsci11101289 34679354 PMC8534220

[5] Mücke M Ludyga S Colledge F Gerber M. Influence of regular physical activity and fitness on stress reactivity as measured with the trier social stress test protocol: a systematic review. Sports Med. (2018) 48(11):2607–22. 10.1007/s40279-018-0979-0 30159718

[6] Salimzadeh R Hall NC Saroyan A. Examining academics’ strategies for coping with stress and emotions: a review of research. Front Educ. (2021) 6. 10.3389/feduc.2021.660676

[7] Strehli I Burns RD Bai Y Ziegenfuss DH Block ME Brusseau TA. Mind–body physical activity interventions and stress-related physiological markers in educational settings: a systematic review and meta-analysis. Int J Environ Res Public Health. (2021) 18(1):224. 10.3390/ijerph18010224 PMC7795448 33396730

[8] Grimani A Aboagye E Kwak L. The effectiveness of workplace nutrition and physical activity interventions in improving productivity, work performance and workability: a systematic review. BMC Public Health. (2019) 19(1):1676. 10.1186/s12889-019-8033-1 31830955 PMC6909496

[9] Lusa S Punakallio A Mänttäri S Korkiakangas E Oksa J Oksanen T Interventions to promote work ability by increasing sedentary workers’ physical activity at workplaces – a scoping review. Appl Ergon. (2020) 82:102962. 10.1016/j.apergo.2019.102962 31568961

[10] Opstoel K Chapelle L Prins FJ De Meester A Haerens L van Tartwijk J Personal and social development in physical education and sports: a review study. Eur Phys Educ Rev. (2020) 26(4):797–813. 10.1177/1356336X19882054

[11] Kayani S Kiyani T Wang J Zagalaz Sánchez ML Kayani S Qurban H. Physical activity and academic performance: the mediating effect of self-esteem and depression. Sustainability. (2018) 10(10):3633. 10.3390/su10103633

[12] Lin Y Gao W. The effects of physical exercise on anxiety symptoms of college students: a meta-analysis. Front Psychol. (2023) 14. 10.3389/fpsyg.2023.1136900 PMC10100500 37063553

[13] Martín-Rodríguez A Gostian-Ropotin LA Beltrán-Velasco AI Belando-Pedreño N Simón JA López-Mora C Sporting mind: the interplay of physical activity and psychological health. Sports. (2024) 12(1):37. 10.3390/sports12010037 38275986 PMC10819297

[14] INJEP. Baromètre DJEPVA sur la jeunesse 2016 - INJEP - Sandra Hoibian Nelly Guisse Colette Maes Isa Aldeghi Pauline Jauneau-Cottet. Available online at: https://injep.fr/publication/barometre-djepva-sur-la-jeunesse-2016/ (cited July 30, 2024).

[15] Hammoudi Halat D Soltani A Dalli R Alsarraj L Malki A. Understanding and fostering mental health and well-being among university faculty: a narrative review. J Clin Med. (2023) 12(13):4425. 10.3390/jcm12134425 37445459 PMC10342374

[16] Ribeiro ÍJS Pereira R Freire IV de Oliveira BG Casotti CA Boery EN. Stress and quality of life among university students: a systematic literature review. Health Prof Educ. (2018) 4(2):70–7. 10.1016/j.hpe.2017.03.002

[17] Robb CA. College student financial stress: are the kids alright? J Fam Econ Issues. (2017) 38(4):514–27. 10.1007/s10834-017-9527-6

[18] Steare T Gutiérrez Muñoz C Sullivan A Lewis G. The association between academic pressure and adolescent mental health problems: a systematic review. J Affect Disord. (2023) 339:302–17. 10.1016/j.jad.2023.07.028 37437728

[19] Wathelet M Vincent C Fovet T Notredame CE Habran E Martignène N Evolution in French University Students’ Mental Health One Month After the First COVID-19 Related Quarantine: Results From the COSAMe Survey. Front Psychiatry 2022 10.3389/fpsyt.2022.868369 PMC9110762 35592379

[20] Rolland F Frajerman A Falissard B Bertschy G Diquet B Marra D. Impact of the first wave of the COVID-19 pandemic on French health students. L’Encéphale. (2023) 49(3):219–26. 10.1016/j.encep.2021.12.004 PMC8813577 35221022

[21] Kupcova I Danisovic L Klein M Harsanyi S. Effects of the COVID-19 pandemic on mental health, anxiety, and depression. BMC Psychol. (2023) 11(1):108. 10.1186/s40359-023-01130-5 37041568 PMC10088605

[22] Sahu P. Closure of universities due to coronavirus disease 2019 (COVID-19): impact on education and mental health of students and academic staff. Cureus. (2020) 12(4):e7541. 10.7759/cureus.7541 32377489 PMC7198094

[23] Wu J Kuan G Lou H Hu X Masri MN Sabo A The impact of COVID-19 on students’ anxiety and its clarification: a systematic review. Front Psychol. (2023) 14. 10.3389/fpsyg.2023.1134703 PMC10484512 37691784

[24] Cielo F Ulberg R Di Giacomo D. Psychological impact of the COVID-19 outbreak on mental health outcomes among youth: a rapid narrative review. Int J Environ Res Public Health. (2021) 18(11):6067. 10.3390/ijerph18116067 34199896 PMC8200066

[25] Li B Ng K Tong X Zhou X Ye J Yu JJ. Physical activity and mental health in children and youth during COVID-19: a systematic review and meta-analysis. Child Adolesc Psychiatry Ment Health. (2023) 17(1):92. 10.1186/s13034-023-00629-4 37468975 PMC10357657

[26] Park AH Zhong S Yang H Jeong J Lee C. Impact of COVID-19 on physical activity: a rapid review. J Glob Health. (2022) 12:05003. 10.7189/jogh.12.05003 35493780 PMC8979477

[27] da Silva Castanheira K Sharp M Otto AR. The impact of pandemic-related worry on cognitive functioning and risk-taking. PLoS One. (2021) 16(11):e0260061. 10.1371/journal.pone.0260061 34793534 PMC8601558

[28] Delgado-Alonso C Valles-Salgado M Delgado-Álvarez A Yus M Gómez-Ruiz N Jorquera M Cognitive dysfunction associated with COVID-19: a comprehensive neuropsychological study. J Psychiatr Res. (2022) 150:40–6. 10.1016/j.jpsychires.2022.03.033 35349797 PMC8943429

[29] Korzeniowski CG. Impact of COVID-19 pandemic on the development of children’s executive functions: implications for school-based interventions. J Biomed Res Environ Sci. (2023) 4(6):1120–35. 10.37871/jbres1776

[30] Mufti S. Neurological and Psychological Effects of COVID-19 on Cognitive Impairment. Philadelphia, Pennsylvania, USA: PCOM Capstone Proj (2021).

[31] Onaps O. Enquête sur la Pratique D’activités Physiques et Sportives et la Sédentarité à L’université. France: Onaps.fr. (2023). Available online at: https://onaps.fr/enquete-sur-la-pratique-dactivites-physiques-et-sportives-et-la-sedentarite-a-luniversite/ (cited July 30, 2024).

[32] Herbert C. Enhancing mental health, well-being and active lifestyles of university students by means of physical activity and exercise research programs. Front Public Health. (2022) 10. 10.3389/fpubh.2022.849093 35548074 PMC9082407

[33] Ministère de l’Education Nationale et de la Jeunesse. Le Sport au Collège. Available online at: https://www.education.gouv.fr/le-sport-au-college-9524 (cited July 30, 2024).

[34] Ministère de l’Education Nationale et de la Jeunesse. Les équipements Sportifs en France - Partenariat Avec les Collectivités Visant à Favoriser L’enseignement de L’éducation Physique et Sportive Dans les établissements du Second Degré. France: The French Ministry of National Education and Youth (2022).

[35] Duarte DFB Libório JR Cavalcante GME de Aquino TL de Carvalho Bezerra L de Aguiar Rocha Martin AL The effects of mindfulness-based interventions in COVID-19 times: a systematic review. J Hum Growth Dev. (2022) 32(2):315–26. 10.36311/jhgd.v32.13313

[36] Witarto BS Visuddho V Witarto AP Bestari D Sawitri B Melapi TAS Effectiveness of online mindfulness-based interventions in improving mental health during the COVID-19 pandemic: a systematic review and meta-analysis of randomized controlled trials. PLoS One. (2022) 17(9):e0274177. 10.1371/journal.pone.0274177 36129900 PMC9491555

[37] Zhang D Lee EKP Mak ECW Ho CY Wong SYS. Mindfulness-based interventions: an overall review. Br Med Bull. (2021) 138(1):41–57. 10.1093/bmb/ldab005 33884400 PMC8083197

[38] Carr-Gomm P. Sophrology - a self-care treasure waiting to be discovered by the wider world. J Holist Healthcare. (2021) 18:28. Available online at: https://openurl.ebsco.com/contentitem/gcd:151579522?sid=ebsco:plink:crawler&id=ebsco:gcd:151579522

[39] Kabat-Zinn J. Mindfulness-based interventions in context: past, present, and future. Clin Psychol Sci Pract. (2003) 10(2):144–56. 10.1093/clipsy.bpg016

[40] NCCIH. Yoga: What You Need To Know. Available online at: https://www.nccih.nih.gov/health/yoga-what-you-need-to-know (cited July 30, 2024).

[41] Tavolacci MP Wouters E Van de Velde S Buffel V Déchelotte P Van Hal G The impact of COVID-19 lockdown on health behaviors among students of a French university. Int J Environ Res Public Health. (2021) 18(8):4346. 10.3390/ijerph18084346 33923943 PMC8072635

[42] Tavolacci MP Ladner J Dechelotte P. COVID-19 pandemic and eating disorders among university students. Nutrients. (2021) 13(12):4294. 10.3390/nu13124294 34959846 PMC8707255

[43] Patin A Ladner J Tavolacci MP. Change in university student health behaviours after the onset of the COVID-19 pandemic. Int J Environ Res Public Health. (2023) 20(1):539. 10.3390/ijerph20010539 PMC9819045 36612861

[44] Matteucci I Corsi M Hurdiel R Pezé T Masson P Porrovecchio A. Health-related behavioral changes during the COVID-19 pandemic. A comparison between cohorts of French and Italian university students. PLOS Glob Public Health. (2023) 3(9):e0002298. 10.1371/journal.pgph.0002298 37682794 PMC10490880

[45] Bouchet-Mayer C Perera É Ferez S. Maintenir, arrêter ou ne pas initier une activité sportive: étude de l’impact des confinements sur la pratique physique des étudiants en France. (2022). Available online at: https://hal.science/hal-03668441 (accessed January 15, 2024).

[46] Goncalves A Le Vigouroux S Charbonnier E. University students’ lifestyle behaviors during the COVID-19 pandemic: a four-wave longitudinal survey. Int J Environ Res Public Health. (2021) 18(17):8998. 10.3390/ijerph18178998 34501605 PMC8430950

[47] Leroy A Wathelet M Fovet T Habran E Granon B Martignène N Mental health among medical, healthcare, and other university students during the first COVID-19 lockdown in France. J Affect Disord Rep. (2021) 6:100260. 10.1016/j.jadr.2021.100260 34746911 PMC8557945

[48] Wathelet M Horn M Creupelandt C Fovet T Baubet T Habran E Mental health symptoms of university students 15 months after the onset of the COVID-19 pandemic in France. JAMA Netw Open. (2022) 5(12):e2249342. 10.1001/jamanetworkopen.2022.49342 36580328 PMC9857035

[49] Wathelet M Duhem S Vaiva G Baubet T Habran E Veerapa E Factors associated with mental health disorders among university students in France confined during the COVID-19 pandemic. JAMA Netw Open. (2020) 3(10):e2025591. 10.1001/jamanetworkopen.2020.25591 33095252 PMC7584927

[50] Gaudel J Ahalli S Fort E Bridai Y Baborier N Charbotel B. The impact of lockdown on mental health in PhD students, a cohort study in a French university. (2023) Available online at: https://hal.science/hal-04139715 (accessed January 26, 2024). 10.1016/j.encep.2022.11.002 37088577

[51] Wathelet M Fovet T Jousset A Duhem S Habran E Horn M Prevalence of and factors associated with post-traumatic stress disorder among French university students 1 month after the COVID-19 lockdown. Transl Psychiatry. (2021) 11. 10.1038/s41398-021-01438-z 34045442 PMC8157529

[52] Husky MM Kovess-Masfety V Swendsen JD. Stress and anxiety among university students in France during COVID-19 mandatory confinement. Compr Psychiatry. (2020) 102:152191. 10.1016/j.comppsych.2020.152191 32688023 PMC7354849

[53] Bourion-Bédès S Tarquinio C Batt M Tarquinio P Lebreuilly R Sorsana C Stress and associated factors among French university students under the COVID-19 lockdown: the results of the PIMS-CoV 19 study. J Affect Disord. (2021) 283:108–14. 10.1016/j.jad.2021.01.041 33540333 PMC7813474

[54] Montero-Marin J Hinze V Mansfield K Slaghekke Y Blakemore SJ Byford S Young people’s mental health changes, risk, and resilience during the COVID-19 pandemic. JAMA Netw Open. (2023) 6(9):e2335016. 10.1001/jamanetworkopen.2023.35016 37733343 PMC10514742

[55] Editorial Perspective: Rapid responses to understand and address children and young people’s mental health in the context of COVID-19. Available online at: https://pubmed.ncbi.nlm.nih.gov/35506327/ (cited November 6, 2024). 10.1111/jcpp.13626 PMC9348194 35506327

[56] Pourghazi F Eslami M Ehsani A Ejtahed HS Qorbani M. Eating habits of children and adolescents during the COVID-19 era: a systematic review. Front Nutr. (2022) 9:1004953. 10.3389/fnut.2022.1004953 36330134 PMC9623566

[57] Apter MJ. Dangerous Edge: The Psychology of Excitement. 1 edn New York: Free Pr (1992). p. 192.

[58] Henneberger PK Cox-Ganser JM. Occupation and COVID-19: lessons from the pandemic. J Allergy Clin Immunol Pract. (2024) 12(8):1997–2007.e2. 10.1016/j.jaip.2024.04.022 38648978 PMC11325298

[59] Nafilyan V Pawelek P Ayoubkhani D Rhodes S Pembrey L Matz M Occupation and COVID-19 mortality in England: a national linked data study of 14.3 million adults. Occup Environ Med. (2022) 79(7):433–41. 10.1136/oemed-2021-107818 34965981

[60] Zhang C Ye M Fu Y Yang M Luo F Yuan J The psychological impact of the COVID-19 pandemic on teenagers in China. J Adolesc Health. (2020) 67(6):747–55. 10.1016/j.jadohealth.2020.08.026 33041204 PMC7543885

[61] Do B Kirkland C Besenyi GM Smock C Lanza K. Youth physical activity and the COVID-19 pandemic: a systematic review. Prev Med Rep. (2022) 29:101959. 10.1016/j.pmedr.2022.101959 36034528 PMC9394097

[62] Mahindru A Patil P Agrawal V. Role of physical activity on mental health and well-being: a review. Cureus. (2023) 15(1):e33475. 10.7759/cureus.33475 36756008 PMC9902068

[63] Matei D Trofin D Iordan DA Onu I Condurache I Ionite C The endocannabinoid system and physical exercise. Int J Mol Sci. (2023) 24(3):1989. 10.3390/ijms24031989 36768332 PMC9916354

[64] [PDF] Mindfulness-based stress reduction (MBSR). | Semantic Scholar. Available online at: https://www.semanticscholar.org/paper/Mindfulness-based-stress-reduction-(MBSR).-Kabat-Zinn/4defbe0fa07bfffceca97ae134f2b73843b61450 (cited August 21, 2024).

[65] Creswell JD. Mindfulness interventions. Annu Rev Psychol. (2017) 68:491–516. 10.1146/annurev-psych-042716-051139 27687118

[66] Goyal M Singh S Sibinga EMS Gould NF Rowland-Seymour A Sharma R Meditation programs for psychological stress and well-being: a systematic review and meta-analysis. JAMA Intern Med. (2014) 174(3):357–68. 10.1001/jamainternmed.2013.13018 24395196 PMC4142584

[67] Wang X Dai Z Zhu X Li Y Ma L Cui X Effects of mindfulness-based stress reduction on quality of life of breast cancer patient: a systematic review and meta-analysis. PLoS One. (2024) 19(7):e0306643. 10.1371/journal.pone.0306643 39028716 PMC11259293

[68] Andrieu B Parry J Porrovecchio A Sirost O, Body Ecology and Emersive Leisure. 1st edn. London, United Kingdom: Routledge; 2019. 250 p.

[69] Presentation. Young People and the Anthropocene. (2019). Available from: https://youngpeopleanthropocene.org/this-website/ (cited September 9, 2024).

[70] Young People and Stories for the Anthropocene. Available online at: https://rowman.com/ISBN/9781538153659 (cited November 5, 2024).

[71] L’AS Rouen UC: Club de Sport à Rouen | ASRUC. Available online at: https://www.asrouenuc.com/as-rouen-uc (cited September 9, 2024).

[72] Dispositif Sport-Santé | I3SP - Institut des sciences du sport-santé de Paris. Available online at: https://i3sp.u-paris.fr/dispositif-sport-sante/ (cited September 9, 2024).

