# Supplementary material for: Stress, physical activity, and mindfulness practices among youth amidst COVID-19
Source: Front Sports Act Living. 2024 Dec 2;6:1493729. doi: 10.3389/fspor.2024.1493729 (PMC11646766; doi:10.3389/fspor.2024.1493729)
Supplement: Supplementary file 1 [file Datasheet1.pdf]

ST1: Overview of SUAPS Directors' Insights on the Development, Impact, and Future of Wellness Activities

Theme	Quotes	Interpretation
Theme 1	"In 2003, this type of activity already existed but in a limited way, these practices were more oriented towards adults, university staff, rather than students."	Initially, SUAPS wellness programs were limited in reach and primarily targeted university staff, reflecting an early recognition of wellness. However, wellness had not yet become a core part of the student experience.
	"Gradually, wellness activities and non-competitive physical activities (non-sporting) have gained more and more importance." "The 2018 decree regarding SUAPS activities calls for the integration of wellness and health activities for students." "The pandemic has accelerated this change and encouraged the practice of wellness."	Over the years, SUAPS Wellness activities have expanded in importance, adopting a more student-centered approach, especially following the regulatory support of the 2018 decree and the increased emphasis on wellness due to the pandemic. These developments highlight SUAPS's commitment to broader health objectives focused on enhancing student well-being.
	"The wellness activities initiative in Rouen was launched by Professor Baguelin." "Two teaching units were established at SUAPS two years ago, one of which is dedicated to wellness and health activities."	Specific initiatives, such as those led by Professor Baguelin and the establishment of dedicated teaching units, highlight SUAPS's intentional efforts to integrate wellness activities into both its academic and extracurricular offerings.
	"For the past 15 years, university sports have primarily consisted of physical and sporting activities with a competitive focus, with competitions organized by the French University Sports Federations." "What is new is the extent to which these activities are now integrated into SUAPS programs."	SUAPS has transitioned from its traditional focus on competitive sports to a more inclusive approach that incorporates wellness activities. This move reflects a university-wide shift toward valuing holistic well-being, making wellness an integral part of SUAPS's mission.
Theme 2	"Activities are selected based on trends, student and staff demand, the reality of the previous year, specifically activities maintained after the spring break, as well as the budget, sports facilities, and available human resources."	The choice of wellness activities is influenced by a variety of factors, including current trends, user demand, and available resources.
	"This offering is developed either in response to explicit demand or based on suggestions from instructors who propose and implement new activities, such as Feldenkrais." "SUAPS then tests these new activities to evaluate user interest and participation."	New wellness offerings are introduced through user requests or instructor proposals and are tested to gauge participant engagement, ensuring they align with users' needs and preferences.

	"Participant feedback and engagement play a key role in the continuation or adjustment of these activities."<br>"This approach allows SUAPS to continuously adapt its program and offer a wide range of activities that meet the needs and expectations of all types of audiences."	Participants' feedback is important in designing ongoing wellness initiatives. This feedback guides decisions on whether to continue, change, or cease activities, ensuring that the program remains aligned with user interests.
	"Next, the SUAPS directors take the necessary steps to find the facilities and personnel capable of successfully carrying out these activities."	The implementation of specified activities requires thorough logistical planning, including acquiring proper facilities and skilled personnel.
Theme 3	"Yes, the pandemic is a factor that has increased the practice of wellness activities within SUAPS."<br>"Following the pandemic, financial and human resources were allocated to improve the wellness activity offerings."<br>"However, when on-site activities resumed, there was a strong desire to offer wellness activities to rebuild social connections and enhance health and well-being."	Directors agreed that the pandemic strengthened the practice of wellness activities at SUAPS, with a greater emphasis on developing social relationships and well-being when in-person activities resumed.
	"After the return of COVID, the range of activities offered by SUAPS did not change."<br>"The same activities were gradually resumed."	Despite the pandemic's impact, SUAPS maintained a consistent range of activities, showing a commitment to stability while adapting as needed.
	"Although some activities experienced slight changes, none were discontinued or replaced by others."<br>"For students, COVID highlighted the benefits of physical practices, with particular attention to wellness activities."	While some activities experienced minor changes, none were fully replaced. However, the epidemic highlighted the benefits of physical activity, particularly wellness habits, for students.
	"During the periods of restrictions, these activities were adapted exclusively for the staff, with remote wellness courses being offered."	During lockdowns, wellness activities were primarily adapted for staff, with virtual courses provided to meet their needs.
Theme 4	"I am not sure that it is necessary to increase the number of wellness activities, but it would be better to make more time slots available."	SUAPS directors reviewed the possibility to expand current wellness offerings by increasing time slots rather than adding new activities, meeting growing demand for existing wellness programs.

	"In the world of federated sports, wellness activities (both mental and physical preparation) are incorporated into the majority of disciplines to maintain good health, relax under pressure, and more".	Wellness activities are increasingly incorporated into competitive sports as essential tools for health maintenance and stress management, highlighting their role beyond general fitness.
	"Many people are giving up competitive sports in favor of wellness-oriented physical activities, such as Nordic walking".	A noticeable trend shows a shift in participant preferences from competitive sports to wellness-focused activities, aligning with a broader interest in holistic well-being.

ST2: Instructor Insights on the Structure, Challenges, and Evolution of SUAPS Wellness Activities

Theme	Quotes	Interpretation
Theme 1	"Dance instructor with training in Brazil and France." "He spent four years studying yoga under André Van Lysebeth, gaining comprehensive training in pranayama (breathing techniques), asanas (postures), meditation, and movement synchronization with breath." "Trained in Pilates at the Leader Fit Pilates Training Center in France, completing all courses from beginner to advanced levels." "Professional sophrology training (2 years)."	SUAPS instructors bring diverse, specialized expertise to the wellness program, having trained in various wellness disciplines in both France and abroad. Their unique backgrounds contribute to a comprehensive approach in wellness education.
	"Additionally, I have completed training in sports sophrology and playful sophrology, aimed at improving workplace quality of life and supporting students."	With specialized training in sophrology and related fields, instructors can focus on mental and physical well-being, offering targeted activities that support the quality of life for students and staff.
Theme 2	"Based on what is discussed, I suggest various movements and visualization exercises." "Includes exercises such as cat-cow, downward-facing dog, and abdominal pose." "I Conduct 6 to 7 different exercises, tailored to various skill levels."	SUAPS wellness sessions are designed to provide a variety of exercises, incorporating yoga-based movements and visualization, and are tailored to suit participants' needs and skill levels for a fully engaging experience.
	"Practice a pure breathing exercise." "I incorporate pranayama exercises to consciously work on breathing."	Breathing exercises, particularly those involving pranayama, play a central role in the sessions. These exercises are essential for fostering conscious breathing, which is key to improving overall well-being.
	"The exercises are progressive." "We start with fundamental exercises, and to increase the intensity, we move on to more complex exercises that engage the muscles and joints more deeply."	The sessions are designed to gradually increase in intensity, beginning with basic exercises and moving to more complicated motions that work multiple muscles and joints.
Theme 3	"COVID did not affect student engagement." "The majority of students attending this class do so to fulfill their mandatory wellness credits." "No change; COVID did not alter students' perspectives on yoga."	The pandemic did not significantly affect student interest or engagement in SUAPS wellness courses, as most students participated to fulfill mandatory credits rather than from increased interest.

	"After the COVID period, the instructor noticed a slight increase in young people's engagement in Pilates classes."	A slight increase in young people’s participation in Pilates classes post-COVID suggests a growing interest in this specific wellness activity.
	"However, adults remained committed to practicing Pilates during this period, showing greater consistency and dedication."	In contrast to younger students, adults demonstrated a steady and strong commitment to Pilates throughout the pandemic, highlighting varied responses to wellness activities across age groups.
Theme 4	"Student engagement in the medium and long term is essential."<br>"Encouraging students to place their phones away from their mats and avoid looking at them during the session can be an important rule to promote concentration and immersion in the practice."	One major challenge in engaging youth is ensuring consistent participation and focus. Instructors address this by implementing guidelines, such as setting phones aside, to enhance attentiveness and encourage a deeper immersion in the sessions.
	"The main challenges in teaching Pilates are continuity and overcoming prejudices."<br>"Maintaining a regular and consistent practice among participants is an ongoing challenge, as is dispelling the stereotypes associated with this discipline."	Instructors often confront stereotypes and misconceptions around wellness practices. These assumptions may deter students from completely committing to regular practice. Overcoming these prejudices is vital for cultivating a good and open attitude towards wellness activities.
	"The Pilates program is often tailored to the participants, taking into account the different levels present in each class."<br>"As soon as the instructor observes the progress of the newcomers, they continue the established program with the more experienced participants, ensuring a harmonious progression for all levels."	Programs are often adapted to accommodate participants’ diverse ability levels, allowing beginners to build skills and more experienced participants to continue progressing, ensuring all students remain engaged regardless of experience."

ST3. Sociodemographic characteristics of the youth group (N=134)

	N	%
**Gender**		
Female	102	76.12
Male	32	23.88
**University**		
Université de Paris Cité	37	27.61
Université de Rouen Normandie	97	72.39
**Faculty**		
Law, Economics, and Management	16	11.94
Arts and Humanities	26	19.40
Health Sciences	15	11.19
Human and Social Sciences	20	14.93
Science and Technology	18	13.43
Science and Techniques of Physical and Sport Activities (STAPS)	12	8.96
Other	27	20.15
**Department** *		
Business and Economics	15	11.28
Languages and Literature	14	10.53
Health and Medicine	16	12.03
Science and Engineering	20	15.04
Humanities and Social Sciences	36	27.07
Science and Techniques of Physical and Sport Activities (STAPS)	10	7.52
Technology and Computer Sciences	2	1.50
Other	20	15.04
**Education Level ***		
BTS	2	1.54
Baccalaureate	1	0.77
Bachelor 1st year	40	30.77
Bachelor 2nd year	26	20.00
Bachelor 3d year	25	19.23
Master 1st year	14	10.77
Master 2nd year	17	13.08
PhD candidate	1	0.77
PhD	4	3.08
**Employment Status**		
Employed	89	66.42
Unemployed	45	33.58

** Missing values*
BTS: Advanced Technician’s Certificate

ST4: Distribution of COVID-19 Positivity by Age Group & Employment Status

Age Group									
COVID Positive	Total Count		Adult		Youth		Senior		
	n	%	N	%	N	%	N	%	Fisher's Test: P-value
**TOTAL**	213	100.00	70	100.00	134	100.00	9	100.00	0.0143*
**0**	71	33.33	13	18.57	55	41.04	3	33.33	0.0143*
**1**	74	34.74	30	42.86	41	30.60	3	33.33	
**2**	52	24.41	20	28.57	31	23.13	1	11.11	
**3 or more**	16	7.51	7	10.00	7	5.22	2	22.22	

Employment Status							
	n	%	N	%	N	%	Chi-Square test: P-value
**TOTAL**	218	100.00	97	100.00	121	100.00	0.0005*
**0**	73	33.49	47	48.45	26	21.49	0.0005*
**1**	75	34.40	27	27.84	48	39.67	
**2**	53	24.31	18	18.56	35	28.93	
**3 ore mroe**	17	7.80	5	5.15	12	9.92	

*P-value < 0.05 indicates statistical significance.

ST5: Distribution of COVID-19 Positivity by education level

COVID Positive	TOTAL Count		BTS		Baccalaureate		PhD		PhD candidate (3d year)		PhD candidate (4th year or more)		HDR	
	n	%	N	%	N	%	N	%	N	%	N	%	N	%
**TOTAL**	205	100.00	5	100.00	8	100.00	17	100.00	3	100.00	14	100.00	1	100.00
**0**	71	34.63	1	20.00	2	25.00	2	11.76			1	7.14		
**1**	69	33.66	4	80.00	4	50.00	8	47.06	1	33.33	6	42.86		
**2**	50	24.39			2	25.00	5	29.41	1	33.33	4	28.57	1	100.00
**3 or more**	15	7.32					2	11.76	1	33.33	3	21.43		

COVID Positive	Bachelor year 1		Bachelor year 2		Bachelor year 3		Master year 1		Master year 2		Fisher Test : P - value
	N	%	N	%	N	%	N	%	N	%	
**TOTAL**	42	100.00	27	100.00	34	100.00	24	100.00	30	100.00	0.0000*
**0**	18	42.86	13	48.15	19	55.88	7	29.17	8	26.67	
**1**	13	30.95	7	25.93	8	23.53	7	29.17	11	36.67	
**2**	10	23.81	6	22.22	3	8.82	8	33.33	10	33.33	
**3 or more**	1	2.38	1	3.70	4	11.76	2	8.33	1	3.33	

*P-value < 0.05 indicates statistical significance

ST6: Distribution of COVID-19 Positivity by Faculty

COVID Positive	TOTAL Count		Other		Law, Economics, and Management		Arts and Humanities		Health Sciences	
	n	%	N	%	N	%	N	%	N	%
**TOTAL**	218	100.00	68	100.00	21	100.00	27	100.00	21	100.00
**0**	73	33.49	20	29.41	9	42.86	10	37.04	7	33.33
**1**	75	34.40	28	41.18	6	28.57	7	25.93	6	28.57
**2**	53	24.31	14	20.59	3	14.29	8	29.63	6	28.57
**3 or more**	17	7.80	6	8.82	3	14.29	2	7.41	2	9.52

COVID Positive	Human and Social Sciences		Science and Technology		Science and Techniques of Physical and Sports Activities (STAPS)		
	N	%	N	%	N	%	Fisher Test: P - value
**TOTAL**	23	100.00	45	100.00	13	100.00	<.0001*
**0**	6	26.09	15	33.33	6	46.15	
**1**	10	43.48	15	33.33	3	23.08	
**2**	3	13.04	15	33.33	4	30.77	
**3 or more**	4	17.39					

*P-value < 0.05 indicates statistical significance

ST7: COVID-19 Positivity and Physical Activity Levels

COVID Positive	TOTAL Count		High		Low		Moderate		Chi-Square test: P-value
	n	%	N	%	N	%	N	%	
**TOTAL**	218	100.00	105	100.00	21	100.00	92	100.00	0.7702
**0**	73	33.49	38	36.19	6	28.57	29	31.52	0.7702
**1**	75	34.40	36	34.29	10	47.62	29	31.52	
**2**	53	24.31	24	22.86	4	19.05	25	27.17	
**3 or more**	17	7.80	7	6.67	1	4.76	9	9.78	

ST8: COVID-19 Positivity and Sport Practice During Lockdown

COVID Positibe	TOTAL Count		No		Yes		Chi-Square test: P-value
	n	%	N	%	N	%	
**TOTAL**	218	100.00	100	100.00	118	100.00	0.9585
**0**	73	33.49	33	33.00	40	33.90	0.9585
**1**	75	34.40	36	36.00	39	33.05	
**2**	53	24.31	24	24.00	29	24.58	
**3 or more**	17	7.80	7	7.00	10	8.47	

ST9: Sports Practice and Wellness Activity Participation Outside of SUAPS

Category	Total Participants		Do not practice sport		Occasionally (Less than once per week)		Regularly (More than once per week)		Chi-Square: P -value
	n	%	N	%	N	%	N	%	
**TOTAL**	218	100.00	76	100.00	62	100.00	80	100.00	0.0000*
No Sports Practice	32	14.68	24	31.58	8	12.90			
Sports Practice	186	85.32	52	68.42	54	87.10	80	100.00	0.0000*
**YOUTH**	134	100.00	42	100.00	40	100.00	52	100.00	0.0000*
No Sports Practice	23	17.16	18	42.86	5	12.50			
Sports Practice	111	82.84	24	57.14	35	87.50	52	100.00	0.0000*

*P-value < 0.05 indicates statistical significance

ST10: Sports Practice and Wellness Activity Duration

Category	Total Participants		Less than 6 months		6 months to 1 year		1 to 3 years		3 to 5 years		More than 5 years		Chi-Square: P -value
	n	%	N	%	N	%	N	%	n	%	N	%	
**TOTAL**	218	100.00	45	100.00	47	100.00	50	100.00	21	100.00	55	100.00	0.0003*
No Sports Practice	32	14.68	16	35.56	6	12.77	5	10.00	2	9.52	3	5.45	
Sports Practice	186	85.32	29	64.44	41	87.23	45	90.00	19	90.48	52	94.55	0.0003*
**YOUTH**	134	100.00	44	100.00	35	100.00	29	100.00	6	100.00	20	100.00	0.0106*
No Sports Practice	23	17.16	15	34.09	5	14.29	2	6.90			1	5.00	
Sports Practice	111	82.84	29	65.91	30	85.71	27	93.10	6	100.00	19	95.00	0.0106*

*P-value < 0.05 indicates statistical significance

ST11: Distribution of Stress Levels by Frequency of Wellness Activity Practice Outside SUAPS

Stress Level	Total Count		No		Yes, occasionally		Yes, regularly		Chi-Square Test: P-value
	n	%	N	%	N	%	N	%	
**TOTAL**	218	100.00	76	100.00	62	100.00	80	100.00	0.0193*
**Low**	50	22.94	14	18.42	8	12.90	28	35.00	
**Moderate**	141	64.68	52	68.42	47	75.81	42	52.50	
**High**	27	12.39	10	13.16	7	11.29	10	12.50	0.0193*

*P-value < 0.05 indicates statistical significance

ST12: Distribution of Stress Levels by Duration of Wellness Practice

Stress Level	Total Count		Less than 6 months		6 months to 1 year		1 to 3 years		3 to 5 years		More than 5 years		Chi-Square: P-value
	n	%	N	%	N	%	N	%	N	%	N	%	
**TOTAL**	218	100.00	45	100.00	47	100.00	50	100.00	21	100.00	55	100.00	0.3660
**Low**	50	22.94	10	22.22	10	21.28	6	12.00	7	33.33	17	30.91	
**Moderate**	141	64.68	29	64.44	33	70.21	35	70.00	11	52.38	33	60.00	
**High**	27	12.39	6	13.33	4	8.51	9	18.00	3	14.29	5	9.09	0.3660

ST13: Distribution of Stress Levels by Wellness Session Duration

Stress Level	Total Count		Less than 10 min		Between 10 & 20 min		Between 20 & 30 min		Between 30 min & 1 h		More than 1 h		Fisher Test: P - value
	n	%	N	%	N	%	N	%	N	%	N	%	
**TOTAL**	116	100.00	63	100.00	21	100.00	13	100.00	9	100.00	10	100.00	0.3463
**Low**	20	17.24	11	17.46	5	23.81	1	7.69	2	22.22	1	10.00	
**Moderate**	82	70.69	46	73.02	14	66.67	10	76.92	7	77.78	5	50.00	
**High**	14	12.07	6	9.52	2	9.52	2	15.38			4	40.00	0.3463

min: minute; h: hou
